# Numerical Processing Impairment in 22q11.2 (LCR22-4 to LCR22-5) Microdeletion: A Cognitive-Neuropsychological Case Study

**DOI:** 10.3389/fpsyg.2018.02193

**Published:** 2018-11-21

**Authors:** Lívia de Fátima Silva Oliveira, Annelise Júlio-Costa, Fernanda Caroline dos Santos, Maria Raquel Santos Carvalho, Vitor Geraldi Haase

**Affiliations:** ^1^Laboratório de Neuropsicologia do Desenvolvimento, Departamento de Psicologia, Universidade Federal de Minas Gerais, Belo Horizonte, Brazil; ^2^Programa de Pós-Graduação em Neurociências, Universidade Federal de Minas Gerais, Belo Horizonte, Brazil; ^3^Programa de Pós-Graduação em Genética, Universidade Federal de Minas Gerais, Belo Horizonte, Brazil; ^4^Departamento de Biologia Geral, Universidade Federal de Minas Gerais, Belo Horizonte, Brazil; ^5^Programa de Pós-graduação em Psicologia, Cognição e Comportamento, Universidade Federal de Minas Gerais, Belo Horizonte, Brazil; ^6^Programa de Pós-graduação em Saúde da Criança e do Adolescente, Universidade Federal de Minas Gerais, Belo Horizonte, Brazil; ^7^Instituto Nacional de Ciência e Tecnologia sobre Comportamento, Cognição e Ensino, São Carlos, Brazil

**Keywords:** math learning difficulties, developmental dyscalculia, 22q11.2DS (LCR22-4 to LCR22-5), cognitive phenotype, Weber fraction, approximate number system

## Abstract

Although progress has been made, the cognitive, biological and, particularly, the genetic underpinnings of math learning difficulties (MD) remain largely unknown. This difficulty stems from the heterogeneity of MD and from the large contribution of environmental factors to its etiology. Understanding endophenotypes, e.g., the role of the Approximate Number System (ANS), may help understanding the nature of MD. MD associated with ANS impairments has been described in some genetic conditions, e.g., 22q11.2 deletion syndrome (22q11.2DS or Velocardiofacial syndrome, VCFS). Recently, a girl with MD was identified in a school population screening. She has a new syndrome resulting from a microdeletion in 22q11.2 (LCR22-4 to LCR22-5), a region adjacent to but not overlapping with region 22q11.2 (LCR22-2 to LCR22-4), typically deleted in VCFS. Here, we describe her cognitive-neuropsychological and numerical-cognitive profiles. The girl was assessed twice, at 8 and 11 years. Her numerical-cognitive performance at both times was compared to demographically similar girls with normal intelligence in a single-case, quasi-experimental study. Neuropsychological assessment was normal, except for relatively minor impairments in executive functions. She presented severe and persistent difficulties in the simplest single-digit calculations. Difficulties in commutative operations improved from the first to the second assessment. Difficulties in subtraction persisted and were severe. No difficulties were observed in Arabic number writing. Difficulties in single-digit calculation co-occurred with basic numerical processing impairments in symbolic and non-symbolic (single-digit comparison, dot sets size comparison and estimation) tasks. Her difficulties suggest ANS impairment. No difficulties were detected in visuospatial/visuoconstructional and in phonological processing tasks. The main contributions of the present study are: (a) this is the first characterization of the neuropsychological phenotype in 22q11.2DS (LCR22-4 to LCR22.5) with normal intelligence; (b) mild forms of specific genetic conditions contribute to persistent MD in otherwise typical persons; (c) heterogeneity of neurogenetic underpinnings of MD is suggested by poor performance in non-symbolic numerical processing, dissociated from visuospatial/visuoconstructional and phonological impairments; (d) similar to what happens in 22q11.2DS (LCR22-2 to LCR22-4), ANS impairments may also characterize 22q11.2DS (LCR22-4 to LCR22-5).

## Introduction

Number processing abilities, such as magnitude comparison and estimation, and knowledge about the numerals, have been implicated in both typical and atypical math learning (Siegler and Braithwaite, 2017). Current discussions focus on the role of accuracy of numerical magnitude representations in the non-symbolic Approximate Number System (ANS) vs. access to these non-symbolic representations through symbolic numbers (Leibovich et al., 2017).

Accuracy in non-symbolic numerical representations has been linked to both typical (Halberda et al., 2008) and atypical (Landerl et al., 2004; Piazza et al., 2010; Mazzocco et al., 2011; Pinheiro-Chagas et al., 2014) math learning. Other studies suggest that symbolic numerical representations play a more important role (Rousselle and Noël, 2007; De Smedt and Gilmore, 2011; Szucs et al., 2013, see review in De Smedt et al., 2013). Meta-analyses indicate that correlations between number processing and math achievement are weak (r's between 0.2 and 0.3) and are slightly larger for symbolic over non-symbolic numerical processing (Chen and Li, 2014; Fazio et al., 2014; Schneider et al., 2017). It is also unknown how and when non-symbolic and symbolic processing influence math learning (Leibovich et al., 2017).

Developmental dyscalculia, math learning difficulties (MD) and number processing impairments have been described for some syndromes of environmental or genetic etiology such as fetal alcohol syndrome (Jacobson et al., 2011), fragile X syndrome in females (Mazzocco, 2001; Villalon-Reina et al., 2013), Turner's syndrome (Bruandet et al., 2004; Zougkou and Temple, 2016), Williams-Beuren syndrome (Krajcsi et al., 2009; Libertus et al., 2014) and velocardiofacial syndrome (VCFS, 22q11.2 deletion syndrome, 22q11.2DS) (Barnea-Goraly et al., 2005; De Smedt et al., 2009; Attout et al., 2017).

The presence of developmental dyscalculia among the phenotypes of many different genetic syndromes suggests that multiple specific genetic factors contribute to the emergence of dyscalculia. As the genotype-phenotype variability of genetic syndromes is large, milder forms of a given syndrome may eventually contribute to MD, particularly in individuals with normal intelligence.

One of the most investigated syndromes associated with developmental dyscalculia is 22q11.2DS, resulting from microdeletions of a specific region on chromosome 22. Chromosome 22 is the second smallest human chromosome and corresponds to approximately 1.6% of human genomic DNA (Genome Reference Consortium, 2018). Genetic alterations on chromosome 22q11.2 have been associated with numerous health conditions, including intellectual disability and schizophrenia. At least 48 genes have been identified in the region associated with 22q11.2DS, including *PRODH* and *COMT*, implicated in cognitive functions through regulation of dopamine metabolism (Karayiorgou et al., 2010; Espe, 2018).

The long arm of chromosome 22 contains interspaced low copy-repeated (LCR) sequences, which make this region susceptible to non-homologous recombination events leading to microdeletions or microduplications. Persons having typical 22q11.2DS present the microdeletion of the 22q11.2 (LCR22-2 to LCR22-4) interval.

To elucidate the genomic variations contributing to math learning difficulties, in a previous population study (*n* = 1,520 children), we investigated some genotypic and phenotypic characteristics of MD children, defined as standardized math achievement below the PR 25 (Ferreira et al., 2012). Among 82 MD children, we identified a 8-year-old girl presenting a microdeletion on chromosome 22q11.2 in the LCR22-4 to LCR22-5 interval (Carvalho et al., 2014).

Reviewing the literature, Carvalho and coworkers characterized a new syndrome, 22q11.2DS (LCR22-4 to LCR22-5), associated with microdeletions spanning only this interval and not extending proximally into the 22q11.2 (LCR22-2 to LCR22-4) interval (typically deleted in 22q11.2DS) or distally, into the 22q11.2 (LCR22-5 to LCR22-6) interval. Further, the authors proposed 22q11.2DS (LCR22-4 to LCR22-5) as an additional cause of dyscalculia in 22q11.2.

22q11.2DS (LCR22-4 to LCR22-5) is characterized by intellectual disability in most cases, and psychiatric symptoms and MD suggesting a heterogeneous condition (Table 1). To date, neither the neuropsychological phenotype nor the impairments in number processing have been detailed. Here, we describe a single-case, quasi-experimental study developed to characterize the cognitive-neuropsychological and numerical-cognitive endophenotypes underlying math learning difficulties in the child having the 22q11.2DS (LCR22-4 to LCR22-5) described by Carvalho et al. (2014).

**Table 1:** Findings in patients with 22q11.2DS spanning exclusively the interval LCR22-4 to LCR22-5.

**Studies**	**Sex**	**Age (year)**	**Gestational alterations**	**Postnatal alterations**	**Physical malformations**	**Cognitive phenotype**	**Behavior problems**	**Specific learning disability**
Saitta et al., 1999	M	2	Prematurity	Normal motor development; speech delay; short stature	Cardiac, velopalatine, bone, facial asymmetry	–	–	–
Mikhail et al., 2007	M	15	Prematurity	No development delay	Bone, facial asymmetry	Inferior visual-motor integration (8.4 years). Intellectual disability	Attention deficit hyperacti-vity disorder (ADHD)	–
Ben-Shachar et al., 2008	M	6	Prematurity	Yes	Cardiac, facial asymmetry, celiac disease	–	No	–
	M	5	Prematurity	No	Facial asymmetry	–	Uncontrolled aggression	–
	M	11	Prematurity	Yes	Velopalatine, bone, facial asymmetry, obesity, karyotype 47,XYY	–	Yes	–
	M	3	Prematurity	Yes	Cardiac, velopalatine, facial asymmetry	–	No	–
	F	3	Prematurity	Yes	Facial asymmetry	–	–	–
	M	4	Prematurity	Yes	Velopalatine, bone, facial asymmetry	–	–	–
Newbern et al., 2008	F	–	–	Restricted posnatal growth	Cardiac, facial asymmetry	Intellectual disability	–	–
	M	–	–	Restricted posnatal growth	Cardiac, velopalatine, boné, facial asymmetry	Intellectual disability	–	–
Rodningen et al., 2008	F	7	Prematurity	Mild psychomotor delay; low muscle tone	Cardiac, bone, facial asymmetry	–	–	–
	M	7	Prematurity	Speech delay	Velopalatine, bone, facial asymmetry	Difficulties in: language comprehension, articulate some sounds, motor tasks	Cooperative person, but he challenges limits set by his parents; good in keep the routines.	–
Xu et al., 2008	M	11 months	Prematurity	–	Cardiac, velopalatine, facial asymmetry	Functioning at a 6–7 months level	–	–
Beaujard et al., 2009	F	35	–	–	Cardiac, velopalatine, facial asymmetry	Intellectual disability	–	–
	M	2 months	Prematurity	–	Cardiac, facial asymmetry	–	–	–
Bruce et al., 2010	F	12	Prematurity	Postnatal growth, motor delay	Cardiac, velopalatine, bone, facial asymmetry	–	–	–
Tan et al., 2011	F	–	Prematurity	Hypotonia	Cardiac, bone, facial asymmetry	–	No	–
Verhoeven et al., 2011	F	18	Prematurity	Psychomotor delay; eating problems	Cardiac, velopalatine, bone, facial asymmetry	Difficulties: planning; concentration; visuospatial perception	Impulsivity mood instability, anxiety; paranoid ideation	Yes. In calculation.
Fagerberg et al., 2012	F	14	Prematurity	–	Cardiac, facial asymmetry	–	Attention déficit	–
	M	13	Prematurity	Global developmental delay	Velopalatine, bone, facial asymmetry	–	–	–
Molck et al., 2013	F	4	Prematurity	Developmental delay: motor delay	Cardiac, bone, facial asymmetry	–	Agitation and attention deficit	–
	F	4	–	Developmental delay	Velopalatine, bone, facial asymmetry	–	–	–
Carvalho et al., 2014 (this study)	F	11	–	–	Velopalatine, bone, facial asymmetry	Normal intelligence; mild executive function deficits	Difficulties in social interaction (social phobia). ADHD	Yes, especially in math and language comprehension
Mikhail et al., 2014	F	7,8	Prematurity	Global developmental delay	Bone, facial asymmetry	Intellectual disability	Talks to oneself and to imaginary friends; social, immaturity	–
	M	9	Prematurity	Global developmental delay	Cardiac, velopalatine, boné, facial asymmetry, seizures	Intellectual disability	Poor impulse control and anger issues	–
	M	20	–	Global developmental delay	Bone, facial asymmetry, seizures, pituitary tumor	Intellectual disability	–	–
	F	10	Prematurity	Global Developmental delay	Cardiac, facial asymmetry	Intellectual disability	ADHD; social immaturity, anxiety; impulsivity	–
	F	20	Prematurity	Global Developmental delay	Seizures	Intellectual disability	ADHD; Asperger Disorder	–
	M	22	Prematurity	Global Developmental delay	Cardiac, facial asymmetry	Intellectual disability	–	–
Lindgren et al., 2015	F	21 months	Prematurity	Growth restriction, motor delay	Cardiac, bone, facial asymmetry	–	–	–
	F	5	Prematurity	Speech and language, speech often unintelligible	Bone, facial asymmetry	Learning disorder, individualized educationnal program	ADHD, pediatric bipolar disorder, aggressive behavior	–
	F (sister)	4	Prematurity	Speech and language, unintelligible speech,	Velopalatine, facial asymmetry	Deficit in visual perception and motor integration, mildly delayed gross motor milestones. Individualized educational programin place	ADHD by DSM-V Oppositional defiant disorder/conduct disorder, aggressive behavior	–
Spineli-Silva et al., 2017	F	11	Prematurity	Speech and developmental delay	Cardiac, velopalatine, bone, facial asymmetry	Intellectual disability	–	–

22q11.2DS (LCR22-4 to LCR22-5) has already been reported in 33 persons (Saitta et al., 1999; Mikhail et al., 2007, 2014; Ben-Shachar et al., 2008; Newbern et al., 2008; Rodningen et al., 2008; Xu et al., 2008; Beaujard et al., 2009; Bruce et al., 2010; Tan et al., 2011; Verhoeven et al., 2011; Fagerberg et al., 2012; Molck et al., 2013; Carvalho et al., 2014; Lindgren et al., 2015; Spineli-Silva et al., 2017).

In general, the published studies describe in broad strokes the phenotypic and genotypic characteristics related to 22q11.2DS (LCR22-4 to LCR22-5) (Table 1), which can be summarized in five topics:

1. Distal microdeletions are a health condition independent of 22q11.2DS. Deletions and duplications in the 22q11.2 region are classified as proximal, central and distal (types I, II and III) (Burnside, 2015). 22q11.2DS (LCR22-4 to LCR22-5) is considered distal type I;
2. Although there is no consensus whether distal microdeletions cause a more subtle (Saitta et al., 1999; Mikhail et al., 2007; Carvalho et al., 2014) or more severe phenotype (Ben-Shachar et al., 2008; Xu et al., 2008; Tan et al., 2011; Lindgren et al., 2015), some characteristics have been described more frequently: (a) congenital heart diseases (most frequently of the Truncus Arteriosus type) observed in 16 of the 32 persons reported in the literature (Saitta et al., 1999; Mikhail et al., 2007, 2014; Ben-Shachar et al., 2008; Newbern et al., 2008; Xu et al., 2008; Beaujard et al., 2009; Bruce et al., 2010; Tan et al., 2011; Verhoeven et al., 2011; Fagerberg et al., 2012; Molck et al., 2013; Spineli-Silva et al., 2017); (b) prematurity and low birth weight reported in almost all patients (Saitta et al., 1999; Mikhail et al., 2007, 2014; Ben-Shachar et al., 2008; Newbern et al., 2008; Rodningen et al., 2008; Xu et al., 2008; Beaujard et al., 2009; Bruce et al., 2010; Tan et al., 2011; Verhoeven et al., 2011; Fagerberg et al., 2012; Molck et al., 2013; Lindgren et al., 2015; Spineli-Silva et al., 2017); (c) language development delay observed in six patients (Saitta et al., 1999; Ben-Shachar et al., 2008; Rodningen et al., 2008; Fagerberg et al., 2012; Lindgren et al., 2015; Spineli-Silva et al., 2017); d) bone malformations reported in 15 patients (Saitta et al., 1999; Mikhail et al., 2007, 2014; Ben-Shachar et al., 2008; Rodningen et al., 2008; Bruce et al., 2010; Tan et al., 2011; Fagerberg et al., 2012; Molck et al., 2013; Spineli-Silva et al., 2017); and, e) facial dysmorphisms marked by micrognathia, microcephaly, narrow palpebral fissures, arched eyebrows, featureless filter, hypertelorism, prominent nose, pointed chin, thin lips, etc. One or more of these phenotypes are reported in at least one patient (Saitta et al., 1999; Mikhail et al., 2007, 2014; Ben-Shachar et al., 2008; Newbern et al., 2008; Rodningen et al., 2008; Xu et al., 2008; Beaujard et al., 2009; Bruce et al., 2010; Tan et al., 2011; Verhoeven et al., 2011; Fagerberg et al., 2012; Molck et al., 2013; Lindgren et al., 2015; Spineli-Silva et al., 2017);
3. There is heterogeneity in intelligence. Most studies have qualitatively characterized intellectual disability. Normal or borderline intelligence is described for some patients (Ben-Shachar et al., 2008; Verhoeven et al., 2011; Carvalho et al., 2014);
4. Behavioral symptoms are briefly cited in most studies: (a) anxiety, social immaturity and social phobia; (b) Attention Deficit Hyperactivity Disorder (ADHD), poor impulse control, anger issues and aggressive behaviors; and, (c) Asperger disorder (Mikhail et al., 2007, 2014; Ben-Shachar et al., 2008; Carvalho et al., 2014; Lindgren et al., 2015);
5. Learning difficulties in mathematics have been reported in two cases with normal or borderline intelligence (Verhoeven et al., 2011; Carvalho et al., 2014). Additionally, Beaujard et al. (2009) described a case with family recurrence in which the mother had a history of learning difficulties.

As mentioned above, developmental dyscalculia is a heterogeneous condition, probably characterized by different subtypes and underlying cognitive mechanisms (Wilson and Dehaene, 2007; Rubinsten and Henik, 2009; Karagiannakis et al., 2014). At least five cognitive mechanisms have been implicated in typical and atypical math achievement: (a) working memory and executive processing, probably associated with ADHD; (b) phonological processing, probably associated with developmental dyslexia; (c) visuospatial and visuoconstructional processing, probably associated with nonverbal learning disability; (d) accuracy of number representations, probably underlying pure cases of developmental dyscalculia; and, eventually, (e) math anxiety, as a compound, aggravating factor.

Number processing deficits in 22q11.2DS (LCR22-4 to LCR22-5) must be contrasted to those observed in typical 22q11.2DS. In the typical 22q11.2DS, two of the most salient cognitive traits associated with developmental dyscalculia are impairments in visuospatial and visuoconstructional processing (Simon et al., 2005a,b; Antshel et al., 2008; Schoch et al., 2014; Wong et al., 2014; Attout et al., 2017), and in the accuracy of non-symbolic and symbolic numerical representations (Simon et al., 2005a,b; De Smedt et al., 2009; Oliveira et al., 2014; Attout et al., 2017; Brankaer et al., 2017). It is not known, for example, whether the numerical and visuospatial processing deficits observed in the typical 22q11.2DS reflect a common underlying impairment or may, eventually, be dissociated. Dissociation between visuospatial and numerical impairments in a case of developmental dyscalculia of genetic origin would be of theoretical interest, and would also hint at the neurobiological systems involved.

So far, no studies have specifically investigated the behavioral and cognitive phenotypes of distal microdeletions in 22q11.2, particularly 22q11.2 (LCR22-4 to LCR22-5). Therefore, the aim of the present study is to investigate and describe in detail the cognitive-neuropsychological characteristics of a girl presenting MD and 22q11.2DS (LCR22-4 to LCR22-5), who was assessed at ages 8 and 11. The underlying assumption is that, although this distal microdeletion is classified as a distinct syndrome, the pattern of general neuropsychological and numerical processing deficits presented by affected persons may resemble that presented by individuals with typical 22q11.2DS. This is based on the observation that some symptoms described for patients having 22q11.2DS (LCR22-4 to LCR22-5) have also been frequently described for patients having typical 22q11.2DS, e.g., conotruncal congenital heart malformations or submucous cleft palate. Therefore, there may be long range effects (Zeitz et al., 2013).

More specifically, we were interested in investigating whether the girl presents impairments in visuospatial and visuoconstructional processing and in the accuracy of numerical representations, two of the most salient phenotypic traits in the typical 22q11.2DS. We were also interested in investigating whether these two forms of impairment are dissociable. To test these hypotheses, we compared her general neuropsychological and numerical-cognitive performance at ages 8 and 11 using a single-case, quasi-experimental design (Crawford et al., 2010).

## Clinical report

A girl with 22q11.2DS (LCR22-4 to LCR22-5), was identified among children in a population screening for math learning difficulties in Belo Horizonte, Brazil (Ferreira et al., 2012; Oliveira-Ferreira et al., 2012; Carvalho et al., 2014). At the time of the screening, she was 8 years old and attending the 3rd grade of elementary school. Her intelligence was normal and her performance on a standardized arithmetic achievement test was below the PR 25. She was then referred for a comprehensive neuropsychological investigation and genotyping. Results of Multiplex Ligation-dependent Probe Amplification (MLPA) indicated the presence of an atypical distal microdeletion on chromosome 22q11.2. This microdeletion was confirmed, and its size was determined through an array CGH (947,631 bp) (Carvalho et al., 2014).

The girl underwent neuropsychological assessment twice. She was initially assessed at 8 years, by occasion of the population screening, and later at 11 years, when attending the 6th grade. She had shown learning difficulties since the beginning of elementary school. According to her mother, the difficulties had always been more severe in mathematics and in the interpretation of texts. She was retained in the 6th grade because of her math difficulties. This happened at the end of the school year, well after the second neuropsychological assessment. There was no history of difficulties in word reading and spelling or initial literacy acquisition. Her favorite subject at school was English and the girl was able to easily learn song lyrics in English.

The parents described her as a shy girl with a tendency to isolate. Additionally, according to them, the girl used to have problems expressing her needs and exposing her difficulties, especially at school. Her only friend was an 18-year-old cousin. She had difficulties initiating social interactions, especially with peers. Eventually, after becoming acquainted, she would interact normally.

At home, the girl was independent and helped with household chores, but performed at a slow pace and had difficulties concentrating in and finishing chores and homework. She was described as hyperactive, inattentive and anxious. The symptoms of hyperactivity were treated with methylphenidate for 2 months. Treatment was discontinued as the symptoms of inattention remained and anxiety symptoms were exacerbated. She had the habit of nail-biting. Parents reported some minor problems related to aggressive behavior. According to them, the girl would occasionally get into fights with her 6-year-old sister.

No information on pregnancy, delivery or initial development was available, as she was adopted at age 1 year. At that time, she was unable to sit or crawl. After 3 months with the adopted family, she began to walk and to utter her first words. Respiratory problems were constant in the first years of life. The parents also reported that occasionally the girl had nocturnal enuresis up to 7 years and a tendency to withhold urine when playing.

She lived with her adoptive parents and a younger sister, enjoying a stable home environment. The parents married 16 years ago. Both parents completed high school and had no history of learning difficulties. The adoptive father had been employed in the same company for more than 25 years. The adoptive mother was a housewife, who had serious health problems related to systemic lupus erythematosus, requiring constant treatment with corticosteroids. Her younger sister was the biological daughter of the couple. Follow-up disclosed that the biological daughter of the couple presented typical school achievement.

On clinical examination, the girl had short stature, normal weight and head circumference, narrow palpebral fissures, long nose, submucosal cleft of the palate, bifid uvula, pointed chin, long and thin fingers, short and broad nails (Carvalho et al., 2014). Her phenotypic characteristics are organized and compared to other published cases in Table 1.

## Methods

The girl participated in a quasi-experimental case study. Her general neuropsychological performance was compared to that of available published Brazilian standards. Numerical-cognitive performance was compared to that of two different but demographically similar groups of typically developing children (Controls) at 8 and 11 years. Typically achieving children participating in the Control group were recruited from public schools and were assessed in the context of the same study in which she was identified. All Controls originated from the same socio-economic background as the girl. Specific statistical procedures were used to compare her performance to that of the Controls (Crawford et al., 2010). At 11 years, she also underwent a psychiatric assessment.

### Participants and procedures

All research procedures complied with the Helsinki principles and were previously approved by the local ethics in research board (Research Ethics Committee of the Federal University of Minas Gerais: CAAE: 0091.0.203.000-10). Informed parental consent was obtained for the purposes of research participation. Informed consent to participate in the study was obtained from the parents in written form and orally from the girl. A specific written consent for publication was also obtained in written form, signed by both the girl and her mother. This informed consent includes their agreement with the publication of the indirectly identifiable information such as gender and age and agreement with the publication of the case report.

All general neuropsychological tests used in the first assessment were reapplied and some tasks were added in the reassessment (Table 2). The same battery of numerical-cognitive tasks was used in the two assessments. At 8 years, the girl's performance in the numerical-cognitive evaluation battery was compared to that of a group of 35 girls (mean age = 8.32 years; *SD* = 0.47 years) attending the 3rd grade of public elementary schools. At 11 years, her performance in the numerical-cognitive evaluation battery was compared to the performance of a group of 24 girls (mean age = 11.38 years; *SD* = 0.49 years) attending the 6th grade of elementary public schools. All the individuals of both Control groups had average intelligence (PRs 50 to 75 on the Raven's Colored Progressive Matrices, Angelini et al., 1999) and did not present learning difficulties as assessed by the TDE Arithmetic and TDE Spelling (Stein, 1994; Oliveira-Ferreira et al., 2012).

**Table 2:** Neuropsychological assessment battery.

**Domain assessed**	**Test**	**References**
Intelligence	Raven's Colored Progressive Matrices	Angelini et al., 1999
	Wechsler Intelligence Scale for Children (WISC-III)	Figueiredo, 2002
School achievement	Brazilian School Achievement Test (TDE)	Stein, 1994; Oliveira-Ferreira et al., 2012
Reading-related abilities	Nonword repetition	Santos and Bueno, 2003
	Nonword Reading	Lopes-Silva et al., 2014
	Phoneme elision	Lopes-Silva et al., 2014
Behavior and psychosocial functioning	Child Behavior Checklist (CBCL)	Rocha et al., 2012
Motor dexterity	9-Hole Peg Test (9-HPT)	Poole et al., 2005
Body representation	Finger localization task	Costa et al., 2011
	Right-left orientation test	Costa et al., 2011
Visuospatial/Visuoconstruc-tional abilities	Rey-Osterrieth Complex Figure copy	Oliveira et al., 2004
Episodic memory	Rey Auditory-verbal Learning Test (RAVLT)	Lacerda, 2012
Short-term and working memory	Digit span	Figueiredo and Nascimento, 2007
	Corsi blocks	Santos et al., 2005
	Consonantal trigrams	Vaz et al., 2010
Executive functions	Semantic word fluency	In house
	5-point design fluency test	In house
	Trail Making Test (TMT) A and B	In house
	Victoria Stroop color-word interference test	Charchat-Fichman and Oliveira, 2009
Numerical-cognitive abilities	Simple reaction time	Costa et al., 2011; Ferreira et al., 2012; Pinheiro-Chagas et al., 2014
	Non-symbolic magnitude comparison task
	Single-digit Magnitude Comparison task
	Set-size Magnitude Estimation
	Arabic number reading task
	Arabic number writing task
	Single-digit operations
	Simple Word Problems

### Instruments

#### Behavioral assessment

At 11 years, her adoptive parents responded the Child Behavior Checklist (CBCL, Rocha et al., 2012), a questionnaire that evaluates behavioral symptoms and psychosocial functioning of individuals aged 6 to 11 years. Her results in the CBCL were compared with the norms for girls of the same age group.

#### General neuropsychological assessment

In Table 2, the general neuropsychological domains evaluated when she was 8 and 11 years old, and their respective tasks and normative references, are summarized.

The Brazilian School Achievement Test (TDE), which was used as a criterion of typicality in school achievement, will be discussed in more detail. The TDE is a standardized test of school performance in children from the 2nd to 7th grades. It comprises three subtests, respectively, of Arithmetic, Reading and Spelling (Stein, 1994; Ferreira et al., 2012). The Arithmetic subtest is composed of three simple verbally presented word problems (i.e., Which is the largest, 28 or 42?) and 35 written arithmetic calculations of increasing complexity (i.e., very easy: 4-1; easy: 1230+150+1620; intermediate: 823^*^96; hard: 3/4+2/8). The single-word Reading subtest of the TDE consists of 70 single-word stimuli, which must be read aloud by the proband. The single-word Spelling subtest consists of dictation of 34 words of increasing syllabic complexity (i.e., toca; balanço; cristalização). The reliability coefficients (Cronbach's α) of the TDE subtests are 0.87 or higher. The TDE has been used in other studies, displaying both reliability and validity in assessing learning difficulties and their cognitive correlates (Moura et al., 2013, 2015; Haase et al., 2014; Lopes-Silva et al., 2014, 2016; Pinheiro-Chagas et al., 2014).

#### Numerical-cognitive assessment

An experimental battery for numerical-cognitive assessment in children and adolescents was used in the present, as well as in previous, studies (Costa et al., 2011; Pinheiro-Chagas et al., 2014). The numerical-cognitive battery comprises tasks of number processing and single-digit calculation. The following tasks were used:

Simple Reaction Time (SRT): The computerized RT task is a visual detection task used to control for possible differences in basic processing speed, not related to numerical tasks. In this task the picture of a wolf (height 9.31 cm; length = 11.59 cm) was displayed in the center of a black screen for a maximum time of 3,000 ms. Participants were instructed to press the spacebar on the keyboard as fast as possible whenever the wolf appeared. Each trial was terminated with the first key press. The task had 30 experimental trials, with an inter-trial interval varying between 2,000 and 8,000 ms. SRT was used to control for eventual effects of general processing speed on the numerical tasks.

Non-symbolic Magnitude Comparison Task: Participants were instructed to compare two sets of black dots, simultaneously presented in two white circles on the left and right sides of the screen. They were required to choose the larger numerosity by pressing a side-congruent key (Pinheiro-Chagas et al., 2014). On each trial, one of the two white circles contained 32 dots (reference numerosity), and the other contained 20, 23, 26, 29, 35, 38, 41, or 44 dots. Each numerosity was presented eight times, and every presentation was arranged in a different spatially pseudo-random configuration. The task comprised 64 testing trials. The maximum stimulus presentation time was 4,000 ms, and the intertrial interval was 700 ms. Between trials, a 3 cm fixation cross appeared on the screen for 500 ms. Non-numerical cues were prevented by using a MATLAB script to design and generate the sets of dots to represent the non-symbolic numerosities (Dehaene et al., 2005). This script was programmed so that, in half of the trials, dot size remained constant and total dot area covaried positively with the numerosity. In the other half of the trials, total dot area remained constant and dot size covaried negatively with numerosity. Each child's data were trimmed to exclude responses ±3 SD away from the individual mean RT. The internal Weber fraction (w) was calculated for each child as an indicator of approximate number system (ANS) or number sense acuity, based on the Log-Gaussian model of number representation (Dehaene, 2007), using the methods described by Piazza et al. (2004).

Single-digit Magnitude Comparison Task: In another task, developed by Pinheiro-Chagas et al. (2014), Arabic digits from 1 to 9 were presented on the computer screen (2.12 cm height, 2.12 cm length). The visual angle of the stimuli vertically and horizontally comprised 2.43°. The children were instructed to compare the stimuli with the reference number 5. The digits were presented in white on a black background. A predefined key on the left side of the keyboard should be pressed with the left hand, if the presented digit was less than 5. If the digit was greater than 5, a right key should be pressed with the right hand. The digit 5 was never presented on the computer screen (internal reference). Numerical distances between the stimuli and the reference digit (5) varied from 1 to 4. Each numerical distance was presented the same number of times. Between trials, a fixation point of the same size and color as the stimuli was presented on the screen. The task comprised 80 experimental trials. The maximum stimulus presentation time was 4,000 ms, and the intertrial interval was 700 ms. Dependent measures were mean accuracy and reaction times. A efficiency score P can also be used as a measure of symbolic magnitude processing efficiency, penalizing RT for inaccuracy: P = RT (1 + 2ER) according to Lyons et al. (2014). In the formula, RT means reaction time and ER stands for error rates, considering reaction time (RT) and errors rates (ER) as measures of performance for each child. ERs were multiplied by 2 because the task was a binary forced choice (ER = 0.5 indicates chance level). Higher scores indicate worse performance. If the performance were perfectly accurate, P would correspond to the individual's average RT (P = RT).

Set-size Magnitude Estimation: In the non-symbolic magnitude estimation task, participants were asked to verbally estimate the quantity of dots shown on the computer screen (Pinheiro-Chagas et al., 2014). The stimuli were black dots presented in a white circle over a black background. The numerosities were 10, 16, 24, 32, 48, 56 or 64 dots. Each numerosity was presented 5 times, each time in a different configuration. The same numerosity never appeared in consecutive trials. The task comprised 35 trials. Counting was avoided by setting the maximum stimulus presentation time to 1000 ms. The examiner, who was seated next to the child, pressed the spacebar and entered the child's response as soon as the child responded. A 3-cm wide/long fixation cross appeared on the screen between individual trials. Use of non-numerical cues was prevented by programming the stimuli in the same manner as those of the non-symbolic number comparison task, described above. Memorization effects due to the repetition of a specific stimulus were avoided in that, in each trial, the stimuli were randomly chosen from a set of 10 precomputed images with the given numerosity. For each subject, data were trimmed to exclude the responses ±3 SD from the mean chosen value across all of the trials. The mean coefficient of variation (cv) was selected as the dependent measure of ANS-accuracy.

Arabic Number Reading Task: Twenty-eight Arabic numbers printed in a booklet were presented, one at a time, and the child had to read the numbers aloud (Moura et al., 2015). The set of items consisted of numbers with up to 4 digits (3 numbers with one digit, 9 numbers with two digits, 8 numbers with three digits and 8 numbers with four digits). The internal consistency of the task is KR-20 = 0.90.

Arabic Number Writing Task: The participant was instructed to write dictated numbers using Arabic numerals (Moura et al., 2015). This task was composed of 40 items, and the numbers contained up to 4 digits (3 numbers with one digit, 9 numbers with two digits, 10 numbers with three digits and 18 numbers with four digits). The internal consistency of the task is 0.96 with the KR-20 formula.

Single-digit operations: This task consisted of single-digit addition (27 items), subtraction (27 items), and multiplication (28 items) operations for individual application, which were printed on separate sheets of paper (Costa et al., 2011). Children were instructed to answer as fast and as accurately as they could; time limit per block was 1 min. Arithmetic operations were organized in two levels of complexity and were presented to the children in separate blocks: one block consisted of simple arithmetic table facts and the other block of more complex problems. Simple additions were defined as those operations having results below 10 (e.g., 3 + 5), while complex additions were those having results between 11 and 17 (e.g., 9 + 5). Tie problems (e.g., 4 + 4) were not used for addition. Simple subtractions were defined as those operations having operands less than 10 (e.g., 9 – 6), while complex subtractions were defined as those having operands ranging from 11 to 17 (e.g., 16 – 9). No negative results were included in the subtraction problems. Simple multiplications were defined as those operations having results less than 25 and/or with the digit 5 as one of the operands (e.g., 2 × 7, 5 × 6), while complex multiplications were defined as those having products ranging from 24 to 72 (6 × 8). Tie problems were not used for multiplication. Reliability coefficients were high (Cronbach's α > 0.90).

Simple Word Problems: Twelve simple arithmetic problems (e.g., “Gabi has 3 reais. Debora has 6 reais. How much do they have together?”) were read aloud by the examiner and simultaneously presented in written form. The child had to solve the problem mentally and write the answer on the paper, with a time limit of 1 min per problem. The dependent variable was the number of correct responses (for more details, see Costa et al., 2011).

### Statistical analysis

**Figure 1:**
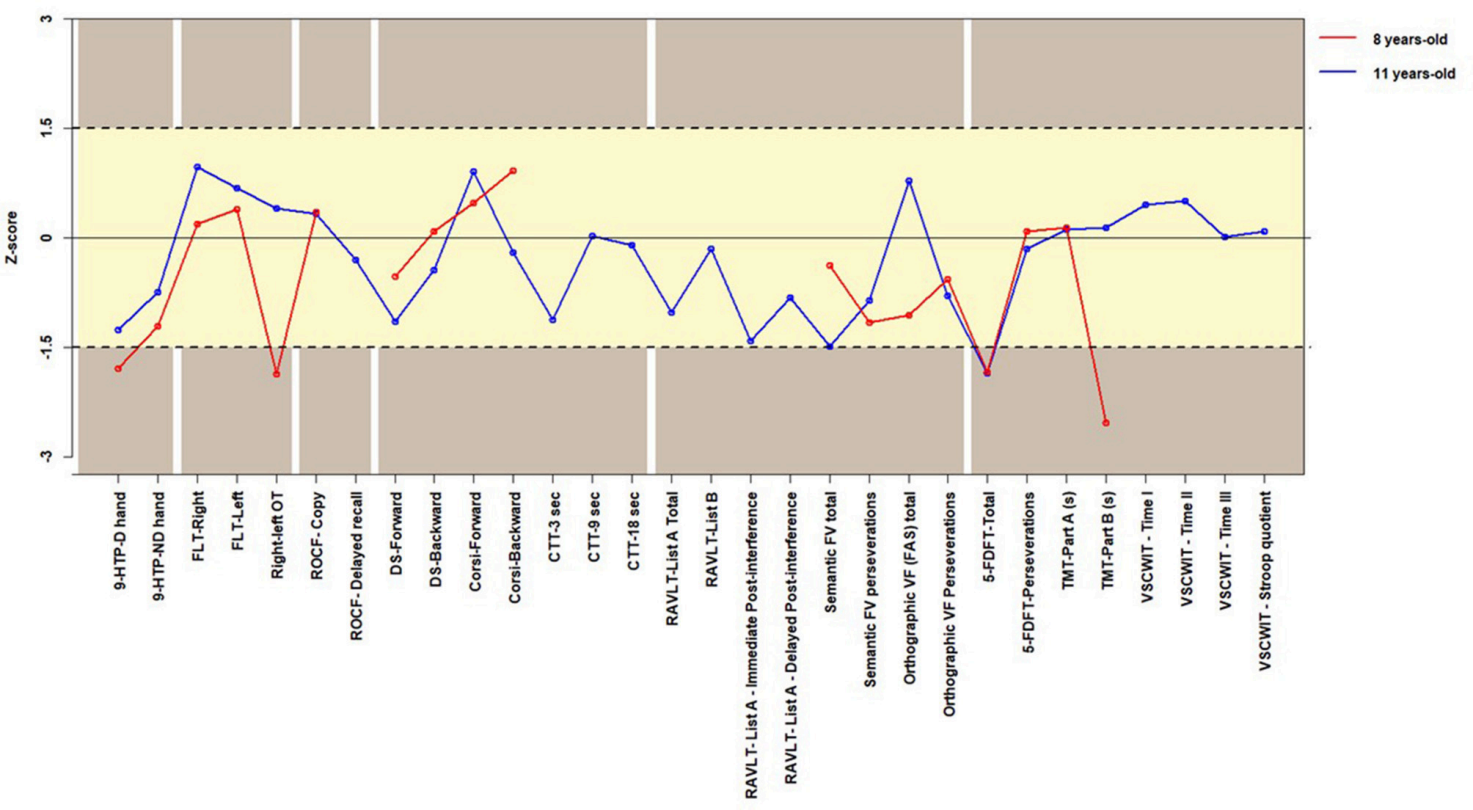
The girl's performance on the general neuropsychological assessment battery. 9-HPT-D.hand, 9-HPT - dominant hand; 9-HPT-ND.hand, 9-HPT - nondominant hand; FLT, finger localization task; ROCF, Rey-Osterrieth Complex Figure; Right-left OT, Right-left Orientation Task Total score; DS, Digit Span; CTT, consonant trigrams test; RAVLT, Rey Auditory-verbal Learning Test; 5-FDFT, 5-point design fluency test; Semantic VF, Semantic Verbal Fluency; Orthographic VF (FAS), Orthographic Verbal Fluency (FAS); VSCWIT, Victoria Stroop color-word interference test.

All scores were z-standardized for age to facilitate comparisons. In the comparison with the published norms, a deviation of 1.5 SD from the mean was used as the cut-off score to determine whether the domain was impaired or preserved. A cut-off score of test performance was employed because diagnosis implies categorization: either the person presents or does not present some health condition. The cut-off score chosen is not overly restrictive or excessively compliant. Larger time executions in the 9-Hole Peg Test (Poole et al., 2005), Trail Making Test, and Victoria Stroop color-word interference test (Charchat-Fichman and Oliveira, 2009) indicate lower performance. Thus, in order to improve their graphic depiction, the direction of change was inverted (Figure 1). The girl's performance on the numerical-cognitive tasks was compared to that of Controls using the statistical methods for neuropsychological case studies developed by Crawford and colleagues (Crawford and Howell, 1998; Crawford and Garthwaite, 2002; Crawford et al., 2010). The analysis concerns the typicality of her performance in comparison with the Control groups. The modified *t*-test proposed by Crawford and Garthwaite (2002) calculated with singlims.exe was used to compare her scores on each task to that of the Control groups' means. Effect size and power analyses were also calculated (Crawford et al., 2010).

## Results

The results are organized into four subsections: intelligence and school performance, behavioral assessment, general neuropsychological assessment and numerical-cognitive assessment.

### Intelligence and school performance

The girl performed normally on intelligence tests, reaching the 60th percentile on Raven's Colored Progressive Matrices (Angelini et al., 1999) in both assessments.

At 11 years, the girl obtained a WISC-III Full-Scale IQ of 98. Although her results did not show a discrepancy between Verbal and Performance IQ, she presented a heterogeneous profile in the subtests.

In the Verbal subscales, the girl obtained average scaled scores in the tasks that involved ability to synthesize and categorize verbal knowledge (Vocabulary: scaled score = 15; z-score = 1.66; Similarities: scaled score = 12; z-score = 0.66; and Comprehension: scaled score = 11; z-score = 0.33). Otherwise, she presented lower scaled scores, still in the normal range, in the tasks that evaluated word problem solving (Arithmetic: scaled score = 8; z-score = −0.66), general knowledge and intellectual curiosity (Information: scaled score = 6; z-score = −1.33), and verbal memory (Digit Span: scaled score = 6; z-score = −1.33). In the performance subscales, the girl obtained average scaled scores in the tasks that involved organization of the whole from separate elements (Object Assembly: scaled score = 12; z-score = 0.66), visual organization (Picture Completion: scaled score = 11; z-score = 0.33), visual memorization and motor coordination (Coding: scaled score = 11; z-score = 0.33). Additionally, she presented below average scaled scores in the tasks that evaluated capacity for visual attention (Symbol Search: scaled score = 6; z-score = −1.33), and analysis and interpretation (Picture Arrangement: scaled score = 4; z-score = −2.00).

The girl's performance on the Spelling and Reading subtests of the TDE (Stein, 1994) was classified as average (PRs between 25 and 75), at both ages of 8 and 11 years. In the TDE Arithmetic subtest, her performance was below the PR 25 at both 8 and 11 years. At 11 years, she was also evaluated with non-word repetition (Santos and Bueno, 2003), non-word reading and phoneme elision tasks (Lopes-Silva et al., 2014). The girl performed at the maximum level in these three phonological processing tasks.

### Behavioral assessment

During the evaluation, the girl was extremely shy and sometimes required extra incentive in order to participate. In the CBCL (Rocha et al., 2012), she attained clinical scores that identified social (*T* = 66), attention (*T* = 73), DSM-anxiety (*T* = 65) and DSM-ADHD problems (*T* = 66). Scores in the other subscales were in the typical range.

### General neuropsychological assessment

At 8 years old, the girl had deficits in motor dexterity in the right (dominant) hand (*z* = −1.79) and in right-left orientation (*z* = −1.87). At 11 years old, her performance on both tasks did not differ from the performance of the Controls in motor dexterity and right-left orientation (Figure 1).

Visuospatial and visuoconstructional abilities were measured using the Rey-Osterrieth Complex Figure copy and delayed recall. The girl performed typically in both evaluations. At 8 years old, her performance was superior to that of the Controls (copy: *z* = 0.35). At 11 years old, her performance was similar to that of the Controls (copy: *z* = 0.32; delayed recall: *z* = −0.31) (Figure 1).

The girl also performed typically on short-term and working memory tasks. On the digit span, her performance was similar to that of the Controls, in both the 8-year-old (z Forward = −0.54; z Backward = 0.08) and 11-year-old (z Forward = −1.15; z Backward = −0.45) evaluations. On the Corsi Blocks, she also performed typically. Her performance was similar to the Controls in both the 8-year-old (z Forward = 0.48; z Backward = 0.92) and 11-year-old (z Forward = 0.90; z Backward = −0.21) evaluations (Figure 1).

At the 11-year-old evaluation, two tasks related to memory were added to the battery of neuropsychological tests. In the Consonantal Trigrams, which evaluate interference in short-term memory, and in the Rey auditory verbal learning test (RAVLT), a task that evaluates verbal long-term memory, the girl performed similarly to the Controls.

The girl presented evidence of impairment in some executive functions in both evaluations. At 8 years old, she. presented low productivity on the 5-point design fluency test, differing from the Controls (*z* = −1.84). This difference persisted in the 11-year-old assessment (*z* = −1.86). Productivity in the semantic word fluency task was typical at 8 years (*z* = −0.38) and slightly over the cut-off score at 11 years (*z* = −1.49). In part B of the Trail Making test, which evaluates motor skills, processing speed, attention capacity (visual search), monitoring, inhibition and set-shifting, she presented a much lower performance than the Controls at the 8-year-old assessment (*z* = −2.54), but no differences were found between the girl and the Controls (*z* = −0.14) at 11 years. At the 11-year-old assessment, one task was added to the battery with the purpose of evaluating the executive functions in more detail. In the Victoria Stroop color-word interference test, which evaluates monitoring, error detection/correction and inhibitory control, she presented satisfactory performance (Stroop quotient: *z* = 0.08).

### Numerical-cognitive abilities

The results of the numerical-cognitive tasks are presented in Table 3. Although the SRT is not a numerical task, it was used to control for effects of general processing speed on numerical tasks. At the 8-year-old assessment, the girl's SRTs were slower than that of the Controls (*p* = 0.01, *d* = 2.33). At the 11-year-old assessment, her performance was similar to that of the Controls (*p* = 0.38, *d* = 0.30). An efficiency score, penalizing reaction time by error rate, was used to index the results in the single-digit magnitude comparison task. No similar compensations were used for speed-accuracy trade-offs in the non-symbolic comparison (w) and set-size magnitude estimation (cv) tasks, as the emphasis on the dependent measures in these tasks is related to accuracy.

**Table 3:** The girl's performance on the numerical-cognitive assessment battery.

**8 years-old**							**11 years-old**					
**Measures**		**Girl**	**Controls (*****n*** = **35)**		***t***	***p***	***d***	**Girl**	**Controls (*****n*** = **24)**		***t***	***p***	***d***
			**Mean**	***SD***					**Mean**	***SD***		
Simple reaction time	Reaction time (ms)	747.69	487.18	112.02	2.29	**0.01**	2.33	388.71	368.93	64.18	0.30	0.38	0.30
Non-symbolic magnitude comparison	Reaction time (ms)	2116.20	1281.65	284.28	2.89	<**0.001**	2.94	1514.72	1117.07	262.04	1.48	0.07	1.51
	Error rate	0.74	0.52	0.11	1.97	**0.02**	2.00	0.49	0.39	0.06	1.63	0.06	1.66
	Weber fraction (w)	–	–	–	–	–	–	0.28	0.20	0.05	1.56	0.06	1.60
Single-digit Magnitude Comparison Task	Reaction time (ms)	1706.94	1041.76	235.50	2.59	**0.01**	2.63	770.62	776.24	197.92	−0.02	0.48	−0.02
	Error rate	0.57	0.35	0.12	1.80	**0.03**	1.83	0.24	0.20	0.05	0.78	0.22	0.80
	Efficiency score P	2715.59	1290.35	337.61	4.16	<**0.001**	4.22	858.19	928.87	280.70	−0.24	0.40	−0.25
Set-size Magnitude Estimation	Coefficient of variation (cv)	–	–	–	–	–	–	0.33	0.14	0.04	4.65	<**0.001**	4.75
Arabic number reading task	Accuracy	20	25.53	3.34	−1.63	**0.05**	−1.66	28	27.67	0.56	0.57	0.28	0.58
Arabic number writing task	% correct	78.57	79.62	21.32	−0.05	0.48	−0.05	100	99.55	1.11	0.39	0.34	0.4
Single-digit operations	Simple addition	2	9.69	3.23	2.34	**0.01**	−2.38	11	11.71	0.46	−1.51	0.07	1.54
	Complex addition	0	7.22	4.02	1.77	**0.04**	−1.80	10	12.71	2.40	−1.1	0.14	−1.12
	Simple subtraction	1	8.53	3.34	2.22	**0.01**	−2.25	5	11.04	1.54	−3.84	<**0.001**	−3.92
	Complex subtraction	0	4.69	4.43	1.04	0.15	−1.06	0	8.58	3.40	−2.47	**0.01**	−2.52
	Simple multiplication	–	–	–	–	–	–	15	13.92	2.51	0.42	0.33	0.43
	Complex multiplication	–	–	–	–	–	–	6	6.04	4.00	−0.01	0.49	−0.01
Simple word problems		4	8.81	2.99	1.50	0.06	−1.61	9	10.46	1.56	−0.91	0.18	−0.93

*Bold value indicates Statistical significance: p < 0.05*.

Non-symbolic magnitude comparison: In addition to her higher reaction time on the control task, the girl presented much lower performance in reaction time on the non-symbolic magnitude comparison task (*p* < 0.001, *d* = 2.94), when compared to the Controls. At 8 years, her error rate in the non-symbolic magnitude comparison task was significatively higher (*p* = 0.02, *d* = 2.00). The log-Gaussian model did not adjust at 8 years, so it was not possible to calculate the internal Weber fraction (Table 3). At 11 years old, her reaction times on the non-symbolic magnitude comparison task were slightly above the cut-off score (*p* = 0.07, *d* = 1.51), when compared to the Controls. The internal Weber fraction was 0.28 (*p* = 0.06, *d* = 1.60).

Single-digit magnitude comparison task: At 8 years, the girl presented significantly higher RTs (*p* = 0.01, *d* = 2.63) and error rates (*p* = 0.03, *d* = 1.83) in the single-digit magnitude comparison tasks, when compared to the Controls. Her efficiency score P was significantly higher than that of the Controls (*p* < 0.001, *d* = 4.22). No significant RT (*p* = 0.48, *d* = −0.02), error rate (*p* = 0.22, *d* = 0.80) or efficiency score P (*p* = 0.40, *d* = −0.25) differences were observed at 11 years in the single-digit magnitude comparison task.

Set-size estimation: At 8 years, her performance on the set-size estimation task was random. At 11 years, she presented a significantly higher coefficient of variation when compared to the Controls on the set-size estimation task (*p* = < 0.001, *d* = 4.75).

Single-digit calculation: At 8 years, her performance was lower than that of the Controls on the single-digit operation tasks, both in simple addition (*p* = 0.01, *d* = −2.38) and in simple subtraction (*p* = 0.01, *d* = −1.06). At this age, the girl was unable to perform any slightly more complex addition or subtraction operations. Multiplication items were not applied at 8 years. At 11 years, her performance did not differ from the Controls in simple addition (*p* = 0.07, *d* = 1.54), complex addition (*p* = 0.14, *p* = −1.12), simple multiplication (*p* = 0.33, *d* = 0.43) and complex multiplication (*p* = 0.49, *d* = −0.01) (Table 3). Difficulties in simple subtraction (*p* < 0.001, *d* = −3.92) and complex subtraction (*p* = 0.01, *d* = −2.52) persisted.

Arabic number reading and writing: At 8 years, the girl presented much lower performance than the Controls on the Arabic number reading task (*p* = 0.05, *d* = −1.66). Her performance on the Arabic number writing task was normal (*p* = 0.48, *d* = −0.05). At 11 years, the girl's performance was adequate in tasks that assessed Arabic numbers reading (*p* = 0.28, *d* = 0.58) and writing (*p* = 0.34, *d* = 0.40).

Simple word problems: At 8 years, the girl's performance on simple word problems was below the cut-off score when compared to the Controls (*p* = 0.06, *d* = −1.61). At 11 years, her performance on this task was normal (*p* = 0.18; *d* = −0.93).

## Discussion

This is the first study to characterize in detail the cognitive-neuropsychological phenotype, including cognitive-numerical performance, of an individual with an atypical distal microdeletion on the long arm of chromosome 22 (22q11.2DS LCR22-4 to LCR22-5).The participant is a girl identified through a school population screening for math learning difficulties (Carvalho et al., 2014). This girl was adopted in early infancy and lived in a stable family environment. She was assessed twice, at 8 and 11 years. Her intelligence was normal average at both times. Math learning difficulties persisted from 8 to 11 years, with performance below the PR 25. No difficulties were observed in word reading, word spelling and related phonological abilities. The family reported reading comprehension difficulties. Inattention and social anxiety symptoms were also observed. General neuropsychological assessment disclosed some minor alterations. Visuospatial/visuoconstructional abilities, working memory and long-term memory were average at both times. At 8 years, she exhibited impairments in motor dexterity, right-left orientation and alertness. These impairments were not observed at the 11 years assessment. Difficulties with some executive function tasks were detected at 8 years, such as in the productivity of the 5-point-design fluency task and the set-shifting dimension of the trail-making test. These difficulties had largely disappeared by 11 years.

Persistent math learning difficulties were associated with impairments in both non-symbolic and symbolic numerical magnitude processing and in single-digit calculation. Statistically significant slower reaction times and higher error rates were observed in all non-symbolic and symbolic numerical magnitude processing tasks at 8 years. At 11 years, single-digit magnitude comparison was average, however, she exhibited difficulties with the accuracy of non-symbolic numerical representations (*d* = 1.60) and set-size estimation (*d* = 4.75). Single-digit calculation was consistently impaired at both times. At 11 years, the girl had mastered single-digit addition and multiplication calculations, but she was still struggling with even the most simple subtraction problems. She did not present difficulties with very simple word problems involving single-digit addition and subtraction, at either time. Symbolic numerical transcoding was also typically acquired.

We will discuss the main theoretical and clinical/educational issues raised by the present study in four sections: (a) neuropsychological functioning; (b) cognitive-numerical abilities; (c) mechanisms of math learning difficulties; and (d) clinical and educational implications.

### Neuropsychological functioning

Atypical 22q11.2DS (LCR22-4 a LCR22-5) is a new genetic entity, related but different from typical 22q11.2DS (LCR22-2 a LCR22-4) (Carvalho et al., 2014). Previous research consists exclusively of case (series) reports. The behavioral and cognitive profile of affected individuals was characterized only qualitatively, through clinical description. In this study, we move a step forward, reporting data from a detailed neuropsychological investigation and testing hypothesis regarding the nature of observed cognitive-numerical impairments. We first discuss the results of the general neuropsychologicalassessment.

Intelligence: In general, most cases of 22q11.2DS (LCR22-4 to LCR22-5) have been described as having intellectual disability and receiving special education (Ben-Shachar et al., 2008; Xu et al., 2008; Mikhail et al., 2014; Lindgren et al., 2015). Only one study reported the IQ of a girl with microdeletion in LCR22-4 to LCR22-5 region. In this study, Verhoeven et al. (2011) described a 17-year-old female and her level of intelligence was found to be borderline (total WISC-R IQ = 73). Two cases presenting presumably normal intelligence without detailed description were reported by Ben-Shachar et al. (2008) and Fagerberg et al. (2012).

In children with typical 22q11.2DS, intellectual disability is present in 40% to 45% of affected individuals. When intelligence is normal, usually the IQ is in the borderline range (IQ = 70 to 85, Swillen et al., 1997; Woodin et al., 2001; Green et al., 2009). In children, lower scores are observed in the Performance IQ. This discrepancy tends to decrease in adults (Moberg et al., 2018). One hypothesis is that concomitant lowering of Verbal IQ tends to reduce the discrepance. A reduction of Verbal IQ from childhood to adolescence has been reported in some individuals with typical 22q11.2DS, and it is considered a risk factor for psychosis (Gothelf et al., 2005, 2009).

General intelligence scores remained stable in this girl for three years. Further follow-up is required. Normal intelligence in our participant indicates that intellectual disability is not an necessary phenotypic trait in 22q11.2 (LCR22-4 to LCR22-5). Research on intellectual abilities of individuals with genetic syndromes is biased by the fact that most severe cases have a higher probability of being recognized by families, clinicians and educators.

Visuospatial and motor abilities: Previous reports have underscored the severity of impairments in motor dexterity and visuospatial/visuoconstructional processing in cases of 22q11.2DS (LCR22-4 to LCR22-5). Lindgren et al. (2015) described a 4-year-old patient with 22q11.2DS (LCR22-4 to LCR22-5), that presented deficits in visual perception and motor integration, and mildly delayed gross motor milestones. In 2008, Rodningen and coworkers briefly described a 7-year-old patient with 22q11.2 (LCR22-4 to LCR22-5), presenting the same profile. Additional cases of 22q11.2DS (LCR22-4 to LCR22-5) showing motor deficits have been reported in the literature (Ben-Shachar et al., 2008; Beaujard et al., 2009; Verhoeven et al., 2011; Fagerberg et al., 2012; Mikhail et al., 2014; Spineli-Silva et al., 2017). Impairments in visuomotor integration were reported in two additional articles (Mikhail et al., 2007; Verhoeven et al., 2011).

Individuals with typical 22q11.2DS also present motor delays and difficulties with motor coordination from infancy on (Swillen et al., 1999; Bearden et al., 2001; Gerdes et al., 2001; Vicari et al., 2011). Large and consistent deficits were found for motor skills (d = −1.17) (Moberg et al., 2018). Additionally, occurrence of visuospatial and visuoconstructional impairments is frequent although variable in typical 22q11.2DS (Antshel et al., 2008; Jacobson et al., 2010; Schoch et al., 2014).

Most individuals previously reported with atypical 22q11.2DS were observed in infancy and at preschool age. Unfortunately, as our participant was adopted, there is no information regarding her obstetric and early infancy developmental background. The family reports motor delay at the end of the first year, when she was adopted. This improved in the following 3 months. Minor impairments in motor dexterity, body representation and alertness were observed at 8 years and improved with time (Figure 1). Additionally, and importantly, she did not present visuospatial/visuoconstructional impairments at either time (Figure 1). Anyway, the severity of visuospatial and motor impairments in previous reports of both atypical and typical 22q11.2DS contrast with the mildness of impairments in our participant.

Memory: Memory functions were not investigated in previous reports of atypical 22q11.2DS.

In general, individuals with typical 22q11.2DS present better performance on tasks of verbal rather than visuospatial memory (Woodin et al., 2001; Wong et al., 2014). However, both kinds of memory are impaired compared to controls. Moderate to large effect sizes were found for verbal memory (*d* = −0.70) and visual memory (*d* = −1.0) (Moberg et al., 2018). Individuals with typical 22q11.DS present similar performance as controls in tasks of information acquisition and retrieval (Lajiness-O'Neill et al., 2006; Debbané et al., 2008). Difficulties are more apparent in tasks in which the participant needs to discriminate stimulus relevance. These memory alterations may constitute a trait vulnerability marker signaling increased risk for schizophrenia in the typical 22q11.2DS population (Debbané et al., 2008).

Working and episodic visuospatial memories were intact in our participant (Figure 1). A discrepancy between higher digit span scores and lower but still normal total WISC Digit scores was observed and may be ascribed to attentional fluctuation. Difficulties with attention were also qualitatively observed in the RAVLT performance.

Executive functions: Deficits in executive functions were described in the case of 22q11.2DS (LCR22-4 to LCR22-5) reported by Verhoeven et al. (2011). They described an 18-year-old girl with borderline intelligence and deficits related to planning and concentration. Other reported cases have presented more severe cognitive impairments related to intellectual disability.

Impairments in executive functions are frequent, severe and persistent in individuals with typical 22q11.2DS (Woodin et al., 2001; Robin and Shprintzen, 2005). Moberg et al. (2018) observed moderate to large impairments in basic executive functions (up to *d* = −0.90). Executive function impairments, together with progressive verbal IQ decline, may play a role in the vulnerability to psychiatric disorders, such as psychoses (Gothelf et al., 2005, 2009).

The girl presented difficulties with some executive function tasks. We feel that her deficits in executive functions were slight and tended to improve. In the 3-year period of observation, no deterioration in her cognitive status was observed.

Psychosocial functioning: The girl is the eighth case with 22q11.2DS (LCR22-4 to LCR22-5) reported in the literature presenting symptoms of impulsivity and inattentiveness (Mikhail et al., 2007; Fagerberg et al., 2012). Her psychosocial functioning profile, including attention, social and anxiety problems, had some similarities and differences with those reported previously. Mikhail et al. (2014) described 4 cases with social immaturity, poor impulse control and anger issues, ADHD, anxiety and Asperger Disorder. The girl did not present symptoms of autism, but she presented characteristics of social phobia. Aggressive behaviors also seem to be common in patients with 22q11.2DS (LCR22-4 to LCR22-5) (Ben-Shachar et al., 2008; Verhoeven et al., 2011; Mikhail et al., 2014; Lindgren et al., 2015). Aggressive behavior was not a major issue in the participant.

The relatively mild psychosocial impairment observed in our participant contrasts with the more severe difficulties encountered by individuals with both atypical and typical 22q11.2DS, including the risk of psychosis (Bassett and Chow, 2008). In typical 22q11.2DS, psychosis is estimated to occur in up to 22.6% of patients after adolescence (Bassett and Chow, 2008).

School learning difficulties: Normal intelligence and math learning difficulties have been described in two cases of 22q11.2DS (LCR22-4 to LCR22-5) (Verhoeven et al., 2011; Carvalho et al., 2014). The most salient phenotypic features presented by this participant were the difficulties with number processing and arithmetic calculation. This is the first study to report a detailed neuropsychological investigation of an individual with 22q11.2DS (LCR22-4 to LCR22-5) with normal intelligence and specific learning difficulties.

In summary, the present study suggests a huge variability in the cognitive and behavioral phenotype of 22q11.2DS (LCR22-4 to LCR22-5). Less severely affected individuals may have normal intelligence associated with milder behavioral issues and specific school learning problems. Next, we compare these math learning difficulties with those observed in typical 22q11.2DS (LCR22-2 to LCR22-4). Math learning difficulties will be emphasized, as they are a prominent feature of the present participant as well as in typical 22q11.2DS.

### Cognitive-numerical abilities

It is interesting to compare the profile of cognitive-numerical and arithmetic performance observed in the girl with that of typical 22q11.2DS. Math learning difficulties are a hallmark of the 22q11.2DS phenotype in individuals with normal intelligence (De Smedt et al., 2009). Math learning difficulties in typical 22q11.2DS seem to be unrelated to phonological processing impairments and probably reflect difficulties in more basic numerical and/or visuospatial processing (De Smedt et al., 2008).

De Smedt et al. (2009) observed that 22q11.2DS children's performance did not differ from that of controls in the tasks of reading numbers and single digit calculation. However, 22q11.2DS children were slower than controls in number comparison and in addition/subtraction calculations with larger numbers.

Oliveira et al. (2014) were the first to report inaccuracy of non-symbolic numerical magnitude representations (indexed by the internal Weber fraction, w) in typical 22q11.2DS. However, performance was variable, as not all individuals with 22q11.2DS presented impairments in ANS accuracy. Impairment in ANS, indexed by w in the non-symbolic numerical comparison task, was later confirmed by Attout et al. (2017). Additionally, these authors observed that ANS accuracy was impaired in the visuospatial but not in the auditory version of the non-symbolic comparison task. This suggests a connection between non-symbolic numerical and visuospatial representations. As mentioned before, visuospatial impairments are an important feature of typical 22q11.2DS.

A connection between numerical and spatial representations is suggested by the mental number line model of approximate numerical representations (Dehaene, 1997, 2007; Nieder and Dehaene, 2009). According to this model, the psychophysical signature of numerical magnitude representations suggests a spatialization of approximate numerical representations: (a) numerical magnitude discriminations are increasingly (ratio variability) and proportionally (scalar variability) more difficult as the distance between the numerical stimuli decreases; (b) accuracy in numerical representations also decreases as the numerical magnitude increases in a logarithmically compressed way; finally, (c) smaller digits are processed preferentially by the right and larger digits by the left hemispheres, suggesting a spatial orientation of the mental number line. According to Dehaene (2007) and Nieder and Dehaene (2009), these characteristics indicate that non-symbolic numbers may be represented approximately as a log-Gaussian distribution of the neuronal discharges ordered by numerical magnitudes.

The spatial nature of numerical representations and their impairments in typical 22q11.2DS have been explored in several studies by Simon et al. (2005a,b) and Simon (2008). In these studies, impaired performance of children with 22q11.2DS in a non-symbolic comparison task was associated with visuospatial manipulations reducing stimuli discriminability. According to the granularity hypothesis, Simon (2008) attributed the numerical processing deficits of individuals with 22q11.2DS to a more basic spatial representation inaccuracy or lack of spatial resolution.

Our participant presented persistent math difficulties, investigated from 8 to 11 years. Four possible cognitive-numerical sources for these difficulties may be considered: (a) visuospatial and visuoconstructional impairments; (b) phonological processing impairment; (c) basic numerical impairment; (d) executive dysfunction. The first two are discarded because there was no evidence of impairment in visuospatial/visuoconstructional and phonological processing abilities. Transcoding abilities of more complex numerals is indicative of good spatial and phonological processing abilities. Moreover, improving ability with commutative single-digit operations and persisting difficulties with subtraction suggest an impairment in the ANS. This hypothesis will be considered next.

In the present participant, the agreement among impairments of numerical processing in different modalities and tasks and their persistence is remarkable. Some evidence indicates that experimental tasks of numerical processing lack concurrent validity (Maloney et al., 2010; Price et al., 2012; Pinheiro-Chagas et al., 2014; Smets et al., 2015) and their test-retest reliability has not been explored extensively (Haase et al., 2014). The results indicate that, at least in some cases, basic numerical impairments may be consistent and persistent.

The most remarkable feature of numerical-cognitive impairments in the girl is related to severe impairments in basic numerical magnitude processing. The available data do not allow us to definitely decide if her impairments are related to non-symbolic numerical magnitude representational inaccuracy (Landerl et al., 2004) or to access to non-symbolic representations from symbolic ones (Rousselle and Noël, 2007). Accordingly, an individual could have difficulties learning math owing to some basic numerical magnitude representational deficit or to difficulties with accessing, storing and manipulating numerical information in working memory. These hypotheses will be addressed in the next section, in the context of the mechanisms putatively involved in MD.

### Cognitive mechanisms of math learning difficulties

No substantive qualitative differences were observed in the cognitive mechanisms putatively underlying the present participant's math difficulties and those observed multifactorial developmental math learning difficulties (Wilson and Dehaene, 2007; Karagiannakis et al., 2014). The mathematical behavioral genetic approach partitions variance at the population level and does not allow identification of specific mechanisms implicated in single individuals. This can be accomplished only by molecular-genetic and neuropsychological investigations of specific genetic etiologies.

Current multiple deficit models of developmental disabilities consider that the phenotypic expression is dependent on complex genetic-environmental interactive mechanisms (Pennington, 2006; Johnson, 2012). Relationships between the genetic-environmental etiologic level and the phenotypic expression are not simple, one-to-one, and are subject to environmental sources of regulation at different times. The construct endophenotype was suggested to characterize intermediate steps in this complex, epigenetic path from the genotype to the phenotype (Rutter et al., 2006; Bishop, 2009).

Several endophenotypes were identified in the present study as potentially relevant for the girl's math difficulties as well as for math difficulties in general. In addition to basic numerical processing, discussed in the last section, the following potentially relevant mechanisms were identified in the present participant:

Motor ability: Basic perceptual and motor impairments are a frequent observation in several developmental disorders (Denckla, 1997, 2003), and are predictive of cognitive and behavioral problems at school age (Batstra et al., 2003). Deficits in finger gnosias (Costa et al., 2011) and motor incoordination (Lonnemann et al., 2011) have been described in children with MD. The meaning of these perceptual and motor impairments is uncertain. Bottom-up theories interpret cognitive deficits as a consequence of a disordered developmental process, encompassing the most basic perceptual motor abilities from infancy on (Nicolson and Fawcett, 2010; Elliott and Grigorenko, 2014). According to the procedural deficit hypothesis, MD could be related to difficulties in automatizing the implicit associations underlying numerical concepts and operations (Vandervert, 2017; Prado, 2018). An alternative explanation is that perceptual and motor impairments constitute markers of severity or co-localizares, indicating the presence and anatomic location of brain dysfunction (Denckla, 1997, 2003).

Working memory and executive functions: Impairments in working memory (Raghubar et al., 2010) and executive functions (Bull and Lee, 2014) are an important trait identified in individuals with MD. The ability to store and manipulate information temporarily in working memory is an important requirement at every step in the acquisition of arithmetics, such as counting (Geary et al., 2004), single-digit calculation (Menon et al., 2000; De Smedt et al., 2009), multi-digit calculation (Klein et al., 2009), numerical transcoding, (Barrouillet et al., 2004; Camos, 2008) and word problem solving (Swanson and Sachse-Lee, 2001). Attentional and executive functions have been implied, even in basic quantitative-numerical decisions (Clayton and Gilmore, 2015; Merkley et al., 2016). For example, inhibition of irrelevant perceptual dimensions may play a role in non-symbolic numerical magnitude comparisons. It is notoriously difficult to experimentally control covariation between the discrete numerical and continuous dimensions of stimuli in these tasks (Leibovich and Henik, 2014). The difficulty of the task could then be related to the need to inhibit the irrelevant continuous dimensions, such as surface and luminance, in order to decide based on the relevant discrete magnitude dimension. Other research indicates, however, that in the range of numerosities usually investigated, discrete numerosity is more perceptually salient and associated with math achievement than continuous dimensions such as texture (Anobile et al., 2016).

Math anxiety: Math anxiety is weakly and negatively associated with math achievement, with correlations on the order of −0.25 to −0.40 (Hembree, 1990). Math anxiety is both a risk factor and a consequence of MD (Ma, 1999). However, math anxiety and achievement are dissociable phenomena (Lee, 2009; Stankov et al., 2012), with both highperforming individuals being anxious and lowperforming individuals not being anxious. Usually, math anxiety is not considered a sort of learning disability (Ashcraft and Krause, 2007). It is considered an important concomitant or aggravating factor of existing difficulties.

In summary, several mechanisms were identified as potentially relevant for the MD in the present participant. It is important to balance and to integrate the evidence, connecting it to the big picture of MD in general. It is unfortunate that the genetic and psychosocial background of the participant before adoption is unknown. Data indicates that adopted children have been previously subject to both genetic and environmental risks for poor school achievement (Van Ijzendoorn et al., 2005).

Perceptual and motor impairments and anxiety may also have played a role in the genesis of the girl's math difficulties. Right-left orientation difficulties and motor dexterity improved with time but could have played a role at a crucial moment in learning arithmetics. Math anxiety may have competed for cognitive resources required for math learning at several moments.

The most interesting question is the relative role played by basic numerical processing and executive functioning. The possibility that executive dysfunction may have played a role cannot be excluded. First, her difficulties with executive functions were relatively mild, at least at the times of assessment; and, the clinical history does not suggest severe impairments in self-regulation. Second, her basic numerical processing difficulties were severe, persistent and concordant across modalities and tasks.

The numerical processing abilities of the participant can be interpreted in terms of the criteria proposed by Rousselle and Noël (2007). According to these authors, an access disorder, probably related to executive dysfunction, is characterized by variable and discrepant performance, with sparing of non-symbolic over symbolic numerical processing. The representational deficit is otherwise characterized by modality-independent and comprehensive difficulties with numerical processing. The pervasiveness of the girl's numerical processing difficulties and the mildness of her executive function difficulties suggest a representational deficit.

Investigations at the population and single individual level play complementary roles in partitioning variance and identifying specific sources of difficulties in math achievement. Since working memory and executive function impairments are frequent in all developmental disorders, one important question is related to the specificity of the problem. Why should one kid develop difficulties only in math and the other only in reading?

Multiple deficit models help to understand the complex interplay between specific and general cognitive factors in the origin of MD. According to a model proposed by Johnson (2012), a kid with a basic numerical processing impairment could compensate for the resulting difficulties, if executive processing resources are available. Otherwise, when general processing resources are insufficient, the difficulties are not compensated and may call attention of parents, educators and clinicians, leading to a diagnosis. In the present participant, multiple sources of cognitive and psychosocial variability were identified that could interact with the genetic condition, leading to math learning difficulties.

### Clinical and educational implications

The main results of our study are that math learning difficulties may be associated with a specific genetic etiology (22q11.2DS; LCR22-4 to LCR22-5) and with more or less specific cognitive mechanisms (ANS and/or executive function impairments). Obviously, identifying a potential specific genetic etiology in a case of MD does not ensure that it plays a causal role in the difficulties of that single individual. It also does not exclude a role for other genetic or environmental factors. It is especially important to consider this in the present individual, as the girl was adopted and little information is available on her background before adoption. What are the implications of these findings for neuropsychological and educational practice?

Etiology of developmental and learning disorders is considered to be multifactorial; i.e., resulting from the interaction of several polygenic and environmental influences (Asbury and Plomin, 2013). It is, however, increasingly being recognized that, at the individual level, specific causes may play a role (Carvalho et al., 2014). For example, chromosomal aneuploidies have been recognized as a cause of language development and reading learning difficulties (Simpson et al., 2014). Specific genetic causes also contribute to autism (Cohen et al., 2005). The extreme variability of clinical presentation makes diagnosis difficult in milder cases.

Other research indicates that individuals with learning difficulties present higher rates of medical, especially neurological and psychiatric, comorbidities. This may occur in math (Shalev and Gross-Tsur, 1993) although not in reading learning difficulties (Cuvellier et al., 2004; Billard et al., 2008). Focal cerebral damage has been reported in cases of developmental dyscalculia and dyslexia (Levin et al., 1996; Daigneault and Braun, 2002). Rolandic epilepsy is commonly associated with learning difficulties in children of normal intelligence (Canavese et al., 2007). Common diseases, such as diabetes and asthma, are also more common in children with learning difficulties than in the general population (Blackman and Gurka, 2007; Hannonen et al., 2010).

Specific etiologies might be more common than usually thought. They are not identified because they are not looked for. Polygenes play a causative role at the population but not at the single individual level. The same holds for psychosocial factors. Deprivation, neglect or maltreatment are the most important risk factors for psychopathology and learning difficulties at the population level (Altarac and Saroha, 2007; Belsky, 2007). In a single individual, it is often difficult to establish a causative role for these psychosocial influences, as not all individuals subject to a risk present the outcome (Caspi et al., 2003; Nobile et al., 2010).

Even if the occurrence of a specific etiology were an infrequent event, underdiagnosis has important consequences, as the individual is deprived of proper health and educational counseling. This is especially important in the era of response to intervention (RTI). Learning difficulties are increasingly being handled by teachers in the schools, using the RTI approach, without referral to specialists (Hale et al., 2010). In the RTI approach, it may take several semesters until teachers recognize that a kid presents more severe and stable difficulties that do not respond to the interventions. Furthermore, they may be associated with a higher probability of a genetic etiology. Referrals for specialized diagnosis and care may be delayed for these individuals.

We argue that teachers must be aware of the possibility that children with learning difficulties are a group at risk for several medical, neurological and psychiatric conditions. Our results suggest that math learning difficulties may function as a kind of red-flag, pointing to possible genetic etiologies. Some red-flags for genetic syndromes may be minor, albeit observable by teachers: short or tall stature, congenital malformations, hypotonia, poor motor coordination, anomalous handedness, history of developmental delay, etc. “Funny face” is an important red-flag. These children have no facial malformations but, rather, small, subtle dysmorphisms such as a low nasal bridge, markedly upslanting or downslanting palpebral fissures, small or prominent chin, low set ears, etc. (Huang et al., 2010). Normal people may have one or two such dysmorphisms, but they are not enough to characterize a “funny face.” Minor motor impairments may also hint at a neurological etiology (Daigneault and Braun, 2002; Batstra et al., 2003). Adoption is another important risk factor for developmental disorders of genetic or environmental etiology (Altarac and Saroha, 2007; Tenenbaum et al., 2011). However, it is important not to forget that most children with math learning difficulties will have a perfectly normal constitution and no genetic syndrome.

Finally, our research design has no power to establish a definite role for ANS over executive function impairments in the etiology of the girl's math learning difficulties. Results indicate however, that specific mechanisms, such as ANS and/or executive function impairments vs. phonological and/or visuospatial/visuoconstructional processing, may play a role in specific individuals.

Again, in a given individual, it may difficult to reliably identify which cognitive mechanisms underlie the difficulties. Our own experience has been that, in accordance with the multiple deficits hypothesis, specific and general cognitive impairments interact in complex ways (Haase et al., 2014; Júlio-Costa et al., 2015; Gomides et al., 2018). Identification of the putative mechanisms is relevant for the planning of more efficient interventions (Gomides et al., 2018). Anyway, alone or interacting with general cognitive impairments, ANS may play a role in math learning difficulties. Future research should address the specific mechanisms and crucial developmental period(s) of the ANS involvement with math learning, as well as intervention strategies.

This investigation of a girl with 22q11.2DS (LCR22-4 to LCR22-5), allows us to raise the following points: (a) specific genetic alterations, such as atypical 22q11.2DS, may be related to math learning difficulties in individuals with normal intelligence and slight phenotypic traits that would remain otherwise unrecognized; (b) math learning difficulties may be severe and persistent in these cases, involving both non-symbolic and symbolic numerical magnitude processing, and eventually be associated with executive dysfunctions; (c) although the microdeleted regions in typical and atypical cases of 22q11.2 are non-overlapping, their phenotypic traits may be broadly shared, suggesting long-range interactions and complexity of genotype-phenotype associations (Zeitz et al., 2013); (d) numerical-cognitive impairments were dissociated from spared visuospatial abilities, suggesting heterogeneity of neurogenetic underpinnings. Further studies have the challenge of showing more evidence for these issues.

## Author contributions

VH and MC delineated the study; LO, AJ-C, and VH conducted the neuropsychological evaluation; FS and MC conducted the genetic analyses. All authors contributed in analysing the results and writing the paper. All authors read the final version of the paper and agree with the content of the manuscript.

### Conflict of interest statement

The authors declare that the research was conducted in the absence of any commercial or financial relationships that could be construed as a potential conflict of interest.

## References

[B1] Altarac M. Saroha E. (2007). Lifetime prevalence of learning disability among US children. Pediatrics 119(Suppl. 1), S77–S83. 10.1542/peds.2006-2089L 17272589

[B2] Angelini A. L. Alves I. C. B. Custódio E. M. Duarte W. F. Duarte J. L. M. (1999). Matrizes Progressivas Coloridas de Raven: Escala Especial. Manual. São Paulo: CETEPP.

[B3] Anobile G. Castaldi E. Turi M. Tinelli F. Burr D. C. (2016). Numerosity but not texture-density discrimination correlates with math ability in children. Dev. Psychol. 52:1206 10.1037/dev0000155 27455185 PMC5055099

[B4] Antshel K. M. Fremont W. Kates W. R. (2008). The neurocognitive phenotype in velo-cardio-facial syndrome: a developmental perspective. Dev. Disabil. Res. Rev. 14, 43–51. 10.1002/ddrr.7 18636636

[B5] Asbury K. Plomin R. (2013). G is for Genes: The Impact of Genetics on Education and Achievement. Chichester, UK: Wiley Blackwell 10.1002/9781118482766

[B6] Ashcraft M. H. Krause J. A. (2007). Working memory, math performance, and math anxiety. Psychon. Bull. Rev. 14, 243–248. 10.3758/BF03194059 17694908

[B7] Attout L. Noël M. P. Vossius L. Rousselle L. (2017). Evidence of the impact of visuo-spatial processing on magnitude representation in 22q11. 2 microdeletion syndrome. Neuropsychologia 99, 296–305. 10.1016/j.neuropsychologia.2017.03.023 28342918

[B8] Barnea-Goraly N. Eliez S. Menon V. Bammer R. Reiss A. L. (2005). Arithmetic ability and parietal alterations: a diffusion tensor imaging study in velocardiofacial syndrome. Cogn. Brain Res. 25, 735–740. 10.1016/j.cogbrainres.2005.09.013 16260124

[B9] Barrouillet P. Camos V. Perruchet P. Seron X. (2004). ADAPT: A developmental, asemantic, and procedural model for transcoding from verbal to Arabic numerals. Psychol. Rev. 111, 368–394. 10.1037/0033-295X.111.2.368 15065914

[B10] Bassett A. S. Chow E. W. (2008). Schizophrenia and 22q11.2 deletion syndrome. Curr. Psychiatry Re. 10:148. 10.1007/s11920-008-0026-1 18474208 PMC3129332

[B11] Batstra L. Neeleman J. Hadders-Algra M. (2003). The neurology of learning and behavioural problems in pre-adolescent children. Acta Psychiatr. Scand. 108, 92–100. 10.1034/j.1600-0447.2003.00127.x 12823165

[B12] Bearden C. E. Woodin M. F. Wang P. P. Moss E. McDonald-McGinn D. Zackai E.. (2001). The neurocognitive phenotype of the 22q11. 2 deletion syndrome: selective deficit in visual-spatial memory. J. Clini. Exp. Neuropsychol. 23, 447–464. 10.1076/jcen.23.4.447.1228 11780945

[B13] Beaujard M. P. Chantot S. Dubois M. Keren B. Carpentier W. Mabboux P.. (2009). Atypical deletion of 22q11.2: detection using the FISH TBX1 probe and molecular characterization with high-density SNP arrays. Eur. J. Med. Genet. Year 52, 321–327. 10.1016/j.ejmg.2009.05.010 19467348

[B14] Belsky J. (2007). Experience in childhood and the development of reproductive strategies. Acta Psychol. Sin. 39, 454–468.

[B15] Ben-Shachar S. Ou Z. Shaw C. A. Belmont J. W. Patel M. S. Hummel M. Amato S.. (2008). 22q11.2 distal deletion: a recurrent genomic disorder distinct from DiGeorge syndrome and velocardiofacial syndrome. Am. J. Hum. Genet. 82, 214–221. 10.1016/j.ajhg.2007.09.014 18179902 PMC2253964

[B16] Billard C. Fluss J. Ducot B. Warszawski J. Ecalle J. Magnan A.. (2008). Study of causal factors of reading impairment in a sample of 1062 7 to 8-year-old children. Arch. Pediatrie 15, 1058–1067. 10.1016/j.arcped.2008.02.020 18456475

[B17] Bishop D. V. (2009). Genes, cognition, and communication. Ann. N. Y. Acad. Sci. 1156, 1–18. 10.1111/j.1749-6632.2009.04419.x 19338500 PMC2805335

[B18] Blackman J. A. Gurka M. J. (2007). Developmental and behavioral comorbidities of asthma in children. J. Dev. Behav. Pediatr. 28, 92–99. 10.1097/01.DBP.0000267557.80834.e5 17435459

[B19] Brankaer C. Ghesquière P. De Wel A. Swillen A. De Smedt B. (2017). Numerical magnitude processing impairments in genetic syndromes: a cross-syndrome comparison of Turner and 22q11. 2 deletion syndromes. Dev. Sci. 20:e12458. 10.1111/desc.12458 27748007

[B20] Bruandet M. Molko N. Cohen L. Dehaene S. (2004). A cognitive characterization of dyscalculia in Turner syndrome. Neuropsychologia 42, 288–298. 10.1016/j.neuropsychologia.2003.08.007 14670569

[B21] Bruce S. Hannula-Jouppi K. Puoskari M. Fransson I. Simola K. O. Lipsanen-Nyman M.. (2010). Submicroscopic genomic alterations in Silver–Russell syndrome and Silver–Russell-like patients. J. Med. Genet. 47, 816–822. 10.1136/jmg.2009.069427 19752157

[B22] Bull R. Lee K. (2014). Executive functioning and mathematics achievement. Child Dev. Perspect. 8, 36–41. 10.1111/cdep.12059

[B23] Burnside R. D. (2015). 22q11. 21 deletion syndromes: a review of proximal, central, and distal deletions and their associated features. Cytogenet. Genome Res. 146, 89–99. 10.1159/000438708 26278718

[B24] Camos V. (2008). Low working memory capacity impedes both efficiency and learning of number transcoding in children. J. Exp. Child Psychol. 99, 37–57. 10.1016/j.jecp.2007.06.006 17854821

[B25] Canavese C. Rigardetto R. Viano V. Vittorini R. Bassi B. Pieri I.. (2007). Are dyslexia and dyscalculia associated with Rolandic epilepsy? A short report on ten Italian patients. Epileptic Disord. 9, 432–436. 10.1684/epd.2007.0138 18077230

[B26] Carvalho M. R. S. Vianna G. Oliveira L. F. S. Aguiar M. J. B. Zen P. Haase V. G. (2014). Are 22q11.2 distal deletions associated with math difficulties? Am. J. Med. Genet. A 164, 2256–2262. 10.1002/ajmg.a.36649 24989330

[B27] Caspi A. Sugden K. Moffitt T. E. Taylor A. Craig I. W. Harrington H.. (2003). Influence of life stress on depression: moderation by a polymorphism in the 5-HTT gene. Science 301, 386–389. 10.1126/science.1083968 12869766

[B28] Charchat-Fichman H. Oliveira R. M. (2009). Performance of 119 Brazilian children on Stroop paradigm: victoria version. Arq. Neuropsiquiatr. 67, 445–449. 10.1590/S0004-282X2009000300014 19623442

[B29] Chen Q. Li J. (2014). Association between individual differences in non-symbolic number acuity and math performance: a meta-analysis. Acta Psychol. 148, 163–172. 10.1016/j.actpsy.2014.01.016 24583622

[B30] Clayton S. Gilmore C. (2015). Inhibition in dot comparison tasks. ZDM 47, 759–770. 10.1007/s11858-014-0655-2

[B31] Cohen D. Pichard N. Tordjman S. Baumann C. Burglen L. Excoffier E.. (2005). Specific genetic disorders and autism: clinical contribution towards their identification. J. Autism Dev. Disord. 35, 103–116. 10.1007/s10803-004-1038-2 15796126

[B32] Costa A. J. Lopes-Silva J. G. Pinheiro-Chagas P. Krinzinger H. Lonnemann J. Willmes K.. (2011). A hand full of numbers: a role for offloading in arithmetics learning. Front. Psychol. 2:368. 10.3389/fpsyg.2011.00368 22180748 PMC3235774

[B33] Crawford J. R. Garthwaite P. H. (2002). Investigation of the single case in neuropsychology: Confidence limits on the abnormality of test scores and test score differences. Neuropsychologia 40, 1196–1208. 10.1016/S0028-3932(01)00224-X 11931923

[B34] Crawford J. R. Garthwaite P. H. Porter S. (2010). Point and interval estimates of effect sizes for the case controls design in neuropsychology: Rationale, methods, implementations, and proposed reporting standards. Cogn. Neuropsychol. 27, 245–260. 10.1080/02643294.2010.513967 20936548

[B35] Crawford J. R. Howell D. C. (1998). Comparing an individual's test score against norms derived from small samples. Clin. Neuropsychol. 12, 482–486.

[B36] Cuvellier J. C. Pandit F. Casalis S. Lemaître M. P. Cuisset J. M. Platof A. (2004). Analyse d'une population de 100 enfants adressés pour troubles d'apprentissage scolaire. Archives de Pédiatrie 11, 201–206. 10.1016/j.arcped.2003.12.004 14992765

[B37] Daigneault S. Braun C. M. (2002). Pure severe dyslexia after a perinatal focal lesion: evidence of a specific module for acquisition of reading. J. Dev. & Behav. Pediatr. 23, 256–265. 10.1097/00004703-200208000-00011 12177573

[B38] De Smedt B. Gilmore C. K. (2011). Defective number module or impaired access? Numerical magnitude processing in first graders with mathematical difficulties. J. Exp. Child Psychol. 108, 278–292. 10.1016/j.jecp.2010.09.003 20974477

[B39] De Smedt B. Noël M. P. Gilmore C. Ansari D. (2013). How do symbolic and non-symbolic numerical magnitude processing skills relate to individual differences in children's mathematical skills? A review of evidence from brain and behavior. Trends Neurosci. Educ. 2, 48–55. 10.1016/j.tine.2013.06.001

[B40] De Smedt B. Reynvoet B. Swillen A. Verschaffel L. Boets B. Ghesquière P. (2009). Basic number processing and difficulties in single-digit arithmetic: evidence from velo-cardio-facial syndrome. Cortex 45, 177–188. 10.1016/j.cortex.2007.06.003 19150519

[B41] De Smedt B. Swillen A. Devriendt K. Fryns J. P. Verschaffel L. Boets B.. (2008). Cognitive correlates of mathematical disabilities in children with velo-cardio-facial syndrome. Genet. Couns. 19, 71–94. 18564504

[B42] Debbané M. Glaser B. Eliez S. (2008). Encoding and retrieval processes in velo-cardio-facial syndrome (VCFS). Neuropsychology 22:226. 10.1037/0894-4105.22.2.226 18331165

[B43] Dehaene S. (1997). The Number Sense: How the Mind Creates Mathematics. New York, NY: Oxford University Press.

[B44] Dehaene S. (2007). Symbols and quantities in parietal cortex: elements of a mathematical theory of number representation and manipulation, in Sensoriomotor Foundations of Higher Cognition - Attention and Performance XXII, eds Haggard P. Rossetti Y. Kawato M. (Cambridge: Harvard University Press), 527–574.

[B45] Dehaene S. Izard I. Piazza M. (2005). Control Over Non-Numerical Parameters in Numerosity Experiments. Available online at: www.unicog.org/docs/DocumentationDotsGeneration.doc

[B46] Denckla M. B. (1997). The neurobehavioral examination in children, in Behavioral Neurology and Neuropsychology, eds Feinberg T. E. Farah M. J. (New York, NY: McGraw-Hill), 721–728.

[B47] Denckla M. B. (2003). ADHD: topic update. Brain Dev. 25, 383–389. 10.1016/S0387-7604(03)00057-3 12907270

[B48] Elliott J. G. Grigorenko E. L. (2014). The Dyslexia Debate (No. 14). London: Cambridge University Press.

[B49] Espe S. (2018). MalaCards: the human disease database. J. Med. Library Assoc. 106, 140 10.5195/JMLA.2018.253

[B50] Fagerberg C. R. Graakjaer J. Heinl U. D. Ousager L. B. Dreyer I. Kirchhoff M.. (2012). Heart defects and other features of the 22q11 distal deletion syndrome. Eur. J. Med. Genet. 56, 98–107. 10.1016/j.ejmg.2012.09.009 23063575

[B51] Fazio L. K. Bailey D. H. Thompson C. A. Siegler R. S. (2014). Relations of different types of numerical magnitude representations to each other and to mathematics achievement. J. Exp. Child Psychol. 123, 53–72. 10.1016/j.jecp.2014.01.013 24699178

[B52] Ferreira F. O. Wood G. Pinheiro-Chagas P. Lonnemann J. Krinzinger H. Willmes K. (2012). Explaining school mathematics performance from symbolic and nonsymbolic magnitude processing: similarities and differences between typical and low-achieving children. Psychol. Neurosci. 5, 37–46. 10.3922/j.psns.2012.1.06

[B53] Figueiredo V. L. M. (2002). WISC-III: Escala de Inteligência Wechsler para Crianças. Manual Adaptação e Padronização Brasileira. São Paulo: Casa do Psicólogo.

[B54] Figueiredo V. L. M. Nascimento E. (2007). Desempenhos nas duas tarefas do subteste dígitos do WISC-III e do WAIS-III. Psicologia 23, 313–318. 10.1590/S0102-37722007000300010

[B55] Geary D. C. Hoard M. K. Byrd-Craven J. DeSoto M. C. (2004). Strategy choices in simple and complex addition: Contributions of working memory and counting knowledge for children with mathematical disability. J. Exp. Child Psychol. 88, 121–151. 10.1016/j.jecp.2004.03.002 15157755

[B56] Genome Reference Consortium (2018). Available online at: https://www.ncbi.nlm.nih.gov/grc/human/data.

[B57] Gerdes M. Solot C. Wang P. P. McDonald-McGinn D. M. Zackai E. H. (2001). Taking advantage of early diagnosis: preschool children with the 22q11. 2 deletion. Genet. Med. 3:40. 10.1097/00125817-200101000-00009 11339376

[B58] Gomides M. R. A. Martins G. Z. Starling-Alves I. Júlio-Costa A. Jaeger A. Haase V. G. (2018). Heterogeneity of math difficulties and its implications for interventions in multiplication skills. Dement. Neuropsychol. 12, 256–263. 10.1590/1980-57642018dn12-030006 30425789 PMC6200157

[B59] Gothelf D. Eliez S. Thompson T. Hinard C. Penniman L. Feinstein C.. (2005). COMT genotype predicts longitudinal cognitive decline and psychosis in 22q11. 2 deletion syndrome. Nat. Neurosci. 8:1500. 10.1038/nn1572 16234808

[B60] Gothelf D. Frisch A. Michaelovsky E. Weizman A. Shprintzen R. J. (2009). Velocardiofacial syndrome. J. Ment. Health Res. Intellect. Disabil. 2, 149–167. 10.1080/19315860902756136 20111667 PMC2811959

[B61] Green T. Gothelf D. Glaser B. Debbane M. Frisch A. Kotler M.. (2009). Psychiatric disorders and intellectual functioning throughout development in velocardiofacial (22q11. 2 deletion) syndrome. J. Am. Acad. Child Adolesc. Psychiatry 48, 1060–1068. 10.1097/CHI.0b013e3181b76683 19797984

[B62] Haase V. G. Júlio-Costa A. Lopes-Silva J. B. Starling-Alves I. Antunes A. Pinheiro-Chagas P.. (2014). Contributions from specific and general factors to unique deficits: two cases of mathematics learning difficulties. Front. Psychol. 5:102. 10.3389/fpsyg.2014.00102 24592243 PMC3923187

[B63] Halberda J. Mazzocco M. M. M. Feigenson L. (2008). Individual differences in non-verbal number acuity correlate with maths achievement. Nature 455, 665–668. 10.1038/nature07246 18776888

[B64] Hale J. Alfonso V. Berninger V. Bracken B. Christo C. Clark E. (2010). Critical issues in response-to-intervention, comprehensive evaluation, and specific learning disabilities identification and intervention: an expert white paper consensus. Learn. Disabil. Q. 33, 223–236. 10.1177/073194871003300310

[B65] Hannonen R. Komulainen J. Eklund K. Tolvanen A. Riikonen R. Ahonen T. (2010). Verbal and academic skills in children with early-onset type 1 diabetes. Dev. Med. Child Neurol. 52, e143–e147. 10.1111/j.1469-8749.2010.03648.x 20345954

[B66] Hembree R. (1990). The nature, effects, and relief of mathematics anxiety. J. Res. Math. Educ. 21, 33–46.

[B67] Huang C. J. Chiu H. J. Lan T. H. Wang H. F. Kuo S. W. Chen S. F.. (2010). Significance of morphological features in schizophrenia of a Chinese population. J. Psychiatr. Res. 44, 63–68. 10.1016/j.jpsychires.2009.06.004 19619883

[B68] Jacobson C. Shearer J. Habel A. Kane F. Tsakanikos E. Kravariti E. (2010). Core neuropsychological characteristics of children and adolescents with 22q11. 2 deletion. J. Intell. Disabil. Res. 54, 701–713. 10.1111/j.1365-2788.2010.01298.x 20561146

[B69] Jacobson J. L. Dodge N. C. Burden M. J. Klorman R. Jacobson S. W. (2011). Number processing in adolescents with prenatal alcohol exposure and ADHD: differences in the neurobehavioral phenotype. Alcoholism 35, 431–442. 10.1111/j.1530-0277.2010.01360.x 21158874 PMC3417293

[B70] Johnson M. H. (2012). Executive function and developmental disorders: the flip side of the coins. Trends Cogn. Sci. 16, 454–457. 10.1016/j.tics.2012.07.001 22835639

[B71] Júlio-Costa A. Starling-Alves I. Lopes-Silva J. B. Wood G. Haase V. G. (2015). Stable measures of number sense accuracy in math learning disability: Is it time to proceed from basic science to clinical application? PsyCh J. 4, 218–225. 10.1002/pchj.114 26459122

[B72] Karagiannakis G. Baccaglini-Frank A. Papadatos Y. (2014). Mathematical learning difficulties subtypes classification. Front. Hum. Neurosci. 8:57. 10.3389/fnhum.2014.00057 24574997 PMC3918643

[B73] Karayiorgou M. Simon T. J. Gogos J. A. (2010). 22q11. 2 microdeletions: linking DNA structural variation to brain dysfunction and schizophrenia. Nat. Rev. Neurosci. 11:402. 10.1038/nrn2841 20485365 PMC2977984

[B74] Klein E. Nuerk H. C. Wood G. Knops A. Willmes K. (2009). The exact vs. approximate distinction in numerical cognition may not be exact, but only approximate: How different processes work together in multi-digit addition. Brain Cogn. 69, 369–381. 10.1016/j.bandc.2008.08.031 18929439

[B75] Krajcsi A. Lukács A. Ignács J. Racsmány M. Pléh C. (2009). Numerical abilities in Williams syndrome: dissociating the analogue magnitude system and verbal retrieval. J. Clin. Exp. Neuropsychol. 31, 439–446. 10.1080/13803390802244126 18618357

[B76] Lacerda S. S. (2012). Caracterí*sticas Psicométricas do Teste de Aprendizagem Auditivo-Verbal de Rey e do Teste de Aprendizagem Visual de Desenhos de Rey para a população brasileira*, Unpublished doctoral thesis. Universidade de São Paulo, São Paulo.

[B77] Lajiness-O'Neill R. Beaulieu I. Asamoah A. Titus J. B. Bawle E. Ahmad S.. (2006). The neuropsychological phenotype of velocardiofacial syndrome (VCFS): Relationship to psychopathology. Arch. Clin. Neuropsychol. 21, 175–184. 16307864 10.1016/j.acn.2005.09.001

[B78] Landerl K. Bevan A. Butterworth B. (2004). Developmental dyscalculia and basic numerical capacities: a study of 8–9-year-old students. Cognition 93, 99–125. 10.1016/j.cognition.2003.11.004 15147931

[B79] Lee J. (2009). Universals and specifics of math self-concept, math self-efficacy, and math anxiety across 41 PISA 2003 participating countries. Learn. Individ. Differ. 19, 355–365. 10.1016/j.lindif.2008.10.009

[B80] Leibovich T. Henik A. (2014). Comparing performance in discrete and continuous comparison tasks. Q. J. Exp. Psychol. 67, 899–917. 10.1080/17470218.2013.837940 24070376

[B81] Leibovich T. Katzin N. Harel M. Henik A. (2017). From “sense of number” to “sense of magnitude”: The role of continuous magnitudes in numerical cognition. Behav. Brain Sci. 40:e164 10.1017/S0140525X16000960 27530053

[B82] Levin H. S. Scheller J. Rickard T. Grafman J. Martinkowski K. Winslow M.. (1996). Dyscalculia and dyslexia after right hemisphere injury in infancy. Arch. Neurol. 53, 88–96. 10.1001/archneur.1996.00550010108024 8599565

[B83] Libertus M. E. Feigenson L. Halberda J. Landau B. (2014). Understanding the mapping between numerical approximation and number words: Evidence from Williams syndrome and typical development. Dev. Sci. 17, 905–919. 10.1111/desc.12154 24581047 PMC4150860

[B84] Lindgren V. McRae A. Dineen R. Saulsberry A. Hoganson G. Schrift M. (2015). Behavioral abnormalities are common and severe in patients with distal 22q11. 2 microdeletions and microduplications. Mol. Genet. Genom. Med. 3, 346–353. 10.1002/mgg3.146 26247050 PMC4521969

[B85] Lonnemann J. Linkersdörfer J. Heselhaus V. Hasselhorn M. Lindberg S. (2011). Relations between balancing and arithmetic skills in children–Evidence of cerebellar involvement? J. Neurolinguistics 24, 592–601. 10.1016/j.jneuroling.2011.02.005

[B86] Lopes-Silva J. B. Moura R. Júlio-Costa A. Geraldi Haase V. Wood G. (2014). Phonemic awareness as a pathway to number transcoding. Front. Psychol. 5:13. 10.3389/fpsyg.2014.00013 24478744 PMC3904123

[B87] Lopes-Silva J. B. Moura R. Júlio-Costa A. Wood G. Salles J. F. Haase V. G. (2016). What is specific and what is shared between numbers and words? Front. Psychol. 7:22. 10.3389/fpsyg.2016.00022 26869946 PMC4735706

[B88] Lyons I. M. Price G. R. Vaessen A. Blomert L. Ansari D. (2014). Numerical predictors of arithmetic success in grades 1–6. Dev. Sci. 17, 714–726. 10.1111/desc.12152 24581004

[B89] Ma X. (1999). A meta-analysis of the relationship between anxiety toward mathematics and achievement in mathematics. J. Res. Math. Educ. 30, 520–540.

[B90] Maloney E. A. Risko E. F. Preston F. Ansari D. Fugelsang J. (2010). Challenging the reliability and validity of cognitive measures: the case of the numerical distance effect. Acta Psychol. 134, 154–161. 10.1016/j.actpsy.2010.01.006 20185118

[B91] Mazzocco M. M. (2001). Math learning disability and math LD subtypes: evidence from studies of Turner syndrome, fragile X syndrome, and neurofibromatosis type 1. J. Learn. Disabil. 34, 520–533. 10.1177/002221940103400605 15503567

[B92] Mazzocco M. M. Feigenson L. Halberda J. (2011). Impaired acuity of the approximate number system underlies mathematical learning disability (dyscalculia). Child Dev. 82, 1224–1237. 10.1111/j.1467-8624.2011.01608.x 21679173 PMC4411632

[B93] Menon V. Rivera S. M. White C. D. Glover G. H. Reiss A. L. (2000). Dissociating prefrontal and parietal cortex activation during arithmetic processing. Neuroimage 12, 357–365. 10.1006/nimg.2000.0613 10988030

[B94] Merkley R. Thompson J. Scerif G. (2016). Of huge mice and tiny elephants: exploring the relationship between inhibitory processes and preschool math skills. Front. Psychol. 6:1903. 10.3389/fpsyg.2015.01903 26779057 PMC4703825

[B95] Mikhail F. M. Burnside R. D. Rush B. Ibrahim J. Godshalk R. Rutledge S. L. (2014). The recurrent distal 22q11. 2 microdeletions are often de novo and do not represent a single clinical entity: a proposed categorization system. Genet. Med. 16:92 10.1038/gim.2013.79 23765049

[B96] Mikhail F. M. Descartes M. Piotrowski A. Andersson R. Diaz de Ståhl T. Komorowski J.. (2007). A previously unrecognized microdeletion syndrome on chromosome 22 band q11.2 encompassing the *BCR* gene. Am. J. Med. Genet. 143A, 2178–2184. 10.1002/ajmg.a.31882 17676630

[B97] Moberg P. J. Richman M. J. Roalf D. R. Morse C. L. Graefe A. C. Brennan L. (2018). Neurocognitive functioning in patients with 22q11. 2 deletion syndrome: a meta-analytic review. Behav. Genet. 48, 1–12. 10.1007/s10519-018-9903-5 29922984 PMC7391171

[B98] Molck M. C. Vieira T. P. Sgardioli I. C. Simioni M. Dos Santos A. P. Souza J.. (2013). Atypical copy number abnormalities in 22q11. 2 region: Report of three cases. Eur. J. Med. Genet. 56, 515–520. 10.1016/j.ejmg.2013.07.002 23886712

[B99] Moura R. Lopes-Silva J. B. Vieira L. R. Paiva G. M. de Almeida Prado A. C. Wood G.. (2015). From “Five” to 5 for 5 minutes: arabic number transcoding as a short, specific, and sensitive screening tool for mathematics learning difficulties. Arch. Clin. Neuropsychol. 30, 88–98. 10.1093/arclin/acu071 25488062

[B100] Moura R. J. Wood G. Pinheiro-Chagas P. Lonnemann J. Krinzinger H. Willmes K.. (2013). Transcoding abilities in typical and atypical mathematics achievers: the role of working memory, procedural and lexical competencies. J. Exp. Child Psychol. 116, 707–727. 10.1016/j.jecp.2013.07.008 24007971

[B101] Newbern J. Zhong J. Wickramasinghe R. S. Li X. Wu Y. Samuels I.. (2008). Mouse and human phenotypes indicate a critical conserved role for ERK2 signaling in neural crest development. Proc. Natl. Acad. Sci. U.S.A. 105, 17115–17120. 10.1073/pnas.0805239105 18952847 PMC2579387

[B102] Nicolson R. I. Fawcett A. (2010). Dyslexia, Learning, and the Brain. Cambridge: MIT Press.

[B103] Nieder A. Dehaene S. (2009). Representation of number in the brain. Annu. Rev. Neurosci. 32, 185–208. 10.1146/annurev.neuro.051508.135550 19400715

[B104] Nobile M. Rusconi M. Bellina M. Marino C. Giorda R. Carlet O.. (2010). COMT Val158Met polymorphism and socioeconomic status interact to predict attention deficit/hyperactivity problems in children aged 10–14. Eur. Child Adolesc. Psychiatry 19, 549–557. 10.1007/s00787-009-0080-1 19946720

[B105] Oliveira L. F. Santos A. O. Vianna G. S. Di Ninno C. Q. Giacheti C. M. Carvalho M. R. (2014). Impaired acuity of the approximate number system in 22q11. 2 microdeletion syndrome. Psychol. Neurosci. 7:151 10.3922/j.psns.2014.02.04

[B106] Oliveira M. Rigoni M. Andretta I. Moraes J. F. (2004). Validação do teste figuras complexas de Rey para a população brasileira. Avaliação Psicológica 3, 33–38.

[B107] Oliveira-Ferreira F. Costa D. S. Micheli L. R. Oliveira L. F. S. Pinheiro-Chagas P. Haase V. G. (2012). School Achievement Test: normative data for a representatitive sample of elementar school children. Psychol. Neurosci. 5, 157–164. 10.3922/j.psns.2012.2.05

[B108] Pennington B. F. (2006). From single to multiple deficit models of developmental disorders. Cognition 101, 385–413. 10.1016/j.cognition.2006.04.008 16844106

[B109] Piazza M. Facoetti A. Trussardi A. N. Berteletti I. Conte S. Lucangeli D.. (2010). Developmental trajectory of number acuity reveals a severe impairment in developmental dyscalculia. Cognition 116, 33–41. 10.1016/j.cognition.2010.03.012 20381023

[B110] Piazza M. Izard V. Pinel P. LeBihan D. Dehaene S. (2004). Tuning curves for approximate numerosity in the human parietal cortex. Neuron 44, 547–555. 10.1016/j.neuron.2004.10.014 15504333

[B111] Pinheiro-Chagas P. Wood G. Knops A. Krinzinger H. Lonnemann J. Starling-Alves I.. (2014). In how many ways is the approximate number system associated with exact calculation? PLoS ONE 9:e111155. 10.1371/journal.pone.0111155 25409446 PMC4237330

[B112] Poole J. L. Burtner P. A. Torres T. A. McMullen C. K. Markham A. Marcum M. L.. (2005). Measuring dexterity in children using the Nine-hole Peg Test. J. Hand Ther. 18, 348–351. 10.1197/j.jht.2005.04.003 16059856

[B113] Prado J. (2018). The interplay between learning arithmetic and learning to read: insights from developmental cognitive neuroscience, in Heterogeneity of Function in Numerical Cognition, eds Henik A. Fias W. (Cambridge: Academic Press), 27–49.

[B114] Price G. R. Palmer D. Battista C. Ansari D. (2012). Nonsymbolic numerical magnitude comparison: Reliability and validity of different task variants and outcome measures, and their relationship to arithmetic achievement in adults. Acta Psychol. 140, 50–57. 10.1016/j.actpsy.2012.02.008 22445770

[B115] Raghubar K. P. Barnes M. A. Hecht S. A. (2010). Working memory and mathematics: a review of developmental, individual difference, and cognitive approaches. Learn. Individ. Differ. 20, 110–122. 10.1016/j.lindif.2009.10.005

[B116] Robin N. H. Shprintzen R. J. (2005). Defining the clinical spectrum of deletion 22q11.2. J. Pediatr. 147, 90–96. 10.1016/j.jpeds.2005.03.007 16027702

[B117] Rocha M. M. Rescorla L. A. Emerich D. R. Silvares E. F. M. Borsa J. C. Araújo L. G. S.. (2012). Behavioural/emotional problems in Brazilian children: findings from parents' reports on the child behavior checklist. Epidemiol. Psychiatr. Sci. 22, 329–338. 10.1017/S2045796012000637 23181948 PMC8367334

[B118] Rodningen O. K. Prescott T. Eriksson A. S. Rosby O. (2008). 1.4 Mb recurrent 22q11.2 distal deletion syndrome, two new cases expand the phenotype. Eur. J. Med. Genet. 51, 646–650. 10.1016/j.ejmg.2008.07.007 18725332

[B119] Rousselle L. Noël M. P. (2007). Basic numerical skills in children with mathematics learning disabilities: a comparison of symbolic vs non-symbolic number magnitude processing. Cognition 102, 361–395. 10.1016/j.cognition.2006.01.005 16488405

[B120] Rubinsten O. Henik A. (2009). Developmental dyscalculia: heterogeneity might not mean different mechanisms. Trends Cogn. Sci. 13, 92–99. 10.1016/j.tics.2008.11.002 19138550

[B121] Rutter M. Moffitt T. E. Caspi A. (2006). Gene–environment interplay and psychopathology: multiple varieties but real effects. J. Child Psychol. Psychiatry 47, 226–261. 10.1111/j.1469-7610.2005.01557.x 16492258

[B122] Saitta S. C. McGrath J. M. Mensch H. Shaikh T. H. Zackai E. H. Emanuel B. S. (1999). A 22q11.2 deletion that excludes UFD1L and CDC45L in a patient with conotruncal and craniofacial defects. Am. J. Hum. Genet. 65, 562–566. 10.1086/302514 10417299 PMC1377955

[B123] Santos F. H. Bueno O. F. A. (2003). Validation of the Brazilian children's test of pseudoword repetition in Portuguese speakers aged 4 to 10 years. Braz. J. Med. Biol. Res. 36, 1633–1547. 10.1590/S0100-879X2003001100012 14576909

[B124] Santos F. H. Mello C. B. Bueno O. F. A. Dellatolas G. (2005). Cross-cultural differences for three visual memory tasks in Brazilian children. Percept. Mot. Skills 101, 421–433. 10.2466/pms.101.2.421-433 16383074

[B125] Schneider M. Beeres K. Coban L. Merz S. Susan Schmidt S. Stricker J.. (2017). Associations of non-symbolic and symbolic numerical magnitude processing with mathematical competence: a meta-analysis. Dev. Sci. 20:e12372. 10.1111/desc.12372 26768176

[B126] Schoch K. Harrell W. Hooper S. R. Ip E. H. Saldana S. Kwapil T. R.. (2014). Applicability of the nonverbal learning disability paradigm for children with 22q11. 2 deletion syndrome. J. Learn. Disabil. 47, 153–166. 10.1177/0022219412443556 22572413 PMC4045450

[B127] Shalev R. S. Gross-Tsur V. (1993). Developmental dyscalculia and medical assessment. J. Learn. Disabil. 26, 134–137. 10.1177/002221949302600206 7681863

[B128] Siegler R. S. Braithwaite D. W. (2017). Numerical development. Annu. Rev. Psychol. 68, 187–213. 10.1146/annurev-psych-010416-044101 27687122

[B129] Simon T. J. (2008). A new account of the neurocognitive foundations of impairments in space, time, and number processing in children with chromosome 22q11. 2 deletion syndrome. Dev. Disabil. Res. Rev. 14, 52–58. 10.1002/ddrr.8 18612330 PMC2442464

[B130] Simon T. J. Bearden C. E. Mc-Ginn D. M. Zackai E. (2005a). Visuospatial and numerical cognitive deficits in children with chromosome 22q11. 2 deletion syndrome. Cortex 41, 145–155. 10.1016/S0010-9452(08)70889-X 15714897 PMC4318636

[B131] Simon T. J. Ding L. Bish J. P. McDonald-McGinn D. M. Zackai E. H. Gee J. (2005b). Volumetric, connective, and morphologic changes in the brains of children with chromosome 22q11. 2 deletion syndrome: an integrative study. Neuroimage 25, 169–180. 10.1016/j.neuroimage.2004.11.018 15734353

[B132] Simpson N. H. Addis L. Brandler W. M. Slonims V. Clark A. Watson J.. (2014). Increased prevalence of sex chromosome aneuploidies in specific language impairment and dyslexia. Dev. Med. Child Neurol. 56, 346–353. 10.1111/dmcn.12294 24117048 PMC4293460

[B133] Smets K. Sasanguie D. Szücs D. Reynvoet B. (2015). The effect of different methods to construct non-symbolic stimuli in numerosity estimation and comparison. J. Cogn. Psychol. 27, 310–325. 10.1080/20445911.2014.996568

[B134] Spineli-Silva S. Bispo L. M. Gil-da-Silva-Lopes V. L. Vieira T. P. (2017). Distal deletion at 22q11. 2 as differential diagnosis in Craniofacial Microsomia: case report and literature review. Eur. J. Med. Genet. 61, 262–268. 10.1016/j.ejmg.2017.12.013 29288792

[B135] Stankov L. Lee J. Luo W. Hogan D. J. (2012). Confidence: A better predictor of academic achievement than self-efficacy, self-concept and anxiety? Learn. Individ. Differ. 22, 747–758. 10.1016/j.lindif.2012.05.013

[B136] Stein L. M. (1994). Teste de Desempenho Escolar: Manual para Aplicação e Interpretação. São Paulo: Casa do Psicólogo.

[B137] Swanson H. L. Sachse-Lee C. (2001). Mathematical problem solving and working memory in children with learning disabilities: both executive and phonological processes are important. J. Exp. Child Psychol. 79, 294–321. 10.1006/jecp.2000.2587 11394931

[B138] Swillen A. Devriendt K. Legius E. Eyskens B. Dumoulin M. Gewillig M.. (1997). Intelligence and psychosocial adjustment in velocardiofacial syndrome: a study of 37 children and adolescents with VCFS. J. Med. Genet. 34, 453–458. 10.1136/jmg.34.6.453 9192263 PMC1050966

[B139] Swillen A. Vandeputte L. Cracco J. Maes B. Ghesquière P. Devriendt K.. (1999). Neuropsychological, learning and psychosocial profile of primary school aged children with the velo-cardio-facial syndrome (22q11 deletion): evidence for a nonverbal learning disability? Child Neuropsychol. 5, 230–241. 10.1076/0929-7049(199912)05:04;1-R;FT230 10925707

[B140] Szucs D. Devine A. Soltesz F. Nobes A. Gabriel F. (2013). Developmental dyscalculia is related to visuo-spatial memory and inhibition impairment. Cortex 49, 2674–2688. 10.1016/j.cortex.2013.06.007 23890692 PMC3878850

[B141] Tan T. Y. Collins A. James P. A. McGillivray G. Stark Z. Gordon C. T. (2011). Phenotypic variability of distal 22q11.2 copy number abnormalities. Am. J. Med. Genet. Part A 155, 1623–1633. 10.1002/ajmg.a.34051 21671380

[B142] Tenenbaum A. Talia Dor M. D. Yael Castiel R. N. Sapir A. (2011). Fetal alcohol spectrum disorder in Israel: increased prevalence in an at-risk population. Religion 59:9. 22332440

[B143] Van Ijzendoorn M. H. Juffer F. Poelhuis C. W. K. (2005). Adoption and cognitive development: a meta-analytic comparison of adopted and nonadopted children's IQ and school performance. Psychol. Bull. 131:301. 10.1037/0033-2909.131.2.301 15740423

[B144] Vandervert L. (2017). The origin of mathematics and number sense in the cerebellum: with implications for finger counting and dyscalculia. Cerebell. Ataxias 4:12. 10.1186/s40673-017-0070-x 28748095 PMC5520362

[B145] Vaz I. A. Cordeiro P. M. de Macedo E. C. Lukasova K. (2010). Memória de trabalho em crianças avaliada pela tarefa de Brown-Peterson. Pró-Fono 22, 95–100. 10.1590/S0104-56872010000200005 20640371

[B146] Verhoeven W. Egger J. Brunner H. De Leeuw N. (2011). A patient with a de novo distal 22q11.2 microdeletion and anxiety disorder. Am. J. Med. Genet A 155, 392–397. 10.1002/ajmg.a.33802 21271660

[B147] Vicari S. Mantovan M. Addona F. Costanzo F. Verucci L. Menghini D. (2011). Neuropsychological profile of Italian children and adolescents with 22q11.2 deletion syndrome with and without intellectual disability. Behav. Genet. 42, 287–298. 10.1007/s10519-011-9499-5 21870177

[B148] Villalon-Reina J. Jahanshad N. Beaton E. Toga A. W. Thompson P. M. Simon T. J. (2013). White matter microstructural abnormalities in girls with chromosome 22q11. 2 deletion syndrome, Fragile X or Turner syndrome as evidenced by diffusion tensor imaging. Neuroimage 81, 441–454. 10.1016/j.neuroimage.2013.04.028 23602925 PMC3947617

[B149] Wilson A. J. Dehaene S. (2007). Number sense and developmental dyscalculia. Hum. Behav. Learn. Dev. Brain Atypical Dev. 2, 212–237.

[B150] Wong T. T. Y. Ho C. S. H. Tang J. (2014). Identification of children with mathematics learning disabilities (MLDs) using latent class growth analysis. Res. Dev. Disabil. 35, 2906–2920. 10.1016/j.ridd.2014.07.015 25104225

[B151] Woodin M. Wang P. P. Aleman D. Donald-McGinn D. Zackai E. Moss E. (2001). Neuropsychological profile of children and adolescents with the 22q11.2 microdeletion. Genet. Med. 3, 34–39. 10.1097/00125817-200101000-00008 11339375

[B152] Xu J. Fan Y. S. Siu V. M. (2008). A child with features of Goldenhar syndrome and a novel 1.12 Mb deletion in 22q11.2 by cytogenetics and oligonucleotide array CGH: Is this a candidate region for the syndrome? Am. J. Med. Genet. A 146A, 1886–1889. 10.1002/ajmg.a.32359 18553512

[B153] Zeitz M. J. Lerner P. P. Ay F. Van Nostrand E. Heidmann J. D. Noble W. S.. (2013). Implications of COMT long-range interactions on the phenotypic variability of 22q11. 2 deletion syndrome. Nucleus 4, 487–493. 10.4161/nucl.27364 24448439 PMC3925693

[B154] Zougkou K. Temple C. M. (2016). The processing of number scales beyond whole numbers in development: dissociations in arithmetic in Turner's syndrome. Cogn. Neuropsychol. 33, 277–298. 10.1080/02643294.2016.1179178 27315526

